# LINC01929 mediates breast cancer immunosuppression and is an immunotherapy target

**DOI:** 10.1016/j.isci.2026.115381

**Published:** 2026-03-17

**Authors:** Jaganathan Venkatesh, Marie-Claire D. Wasson, Raj Pranap Arun, Hannah F. Cahill, Olivia L. Walker, Cheryl A. Dean, Wasundara Fernando, Maya R. MacLean, Meghan E. McLean, Bakhmala Khan, Perryn S. Kruth, Mukhayyo Sultonova, Sarah Nersesian, Stacey N. Lee, Modeline N. Longjohn, Christopher S. Hughes, Thomas J. Belbin, Daniel Gaston, Joao A. Paulo, Gregory C. Knapp, Gillian Bethune, Shashi Gujar, Jeanette E. Boudreau, J. Patrick Murphy, Paola Marcato

**Affiliations:** 1 Department of Pathology, Dalhousie University, Halifax, NS, Canada; 2 Beatrice Hunter Cancer Research Institute, Halifax, NS, Canada; 3 Department of Biology, Acadia University, Wolfville, NS, Canada; 4 Department of Biology, University of Prince Edward Island, Charlottetown, PEI, Canada; 5 Department of Microbiology and Immunology, Dalhousie University, Halifax, NS, Canada; 6 Division of Biomedical Sciences, Memorial University of Newfoundland, St. John’s, NL, Canada; 7 Biological Mass Spectrometry Core Facility, Dalhousie University, Halifax, NS, Canada; 8 Discipline of Oncology, Memorial University of Newfoundland, St. John’s, NL, Canada; 9 Nova Scotia Health Authority, Halifax, NS, Canada; 10 Department of Cell Biology, Harvard Medical School, Boston, MA, USA; 11 Department of Surgery, Dalhousie University, Halifax, NS, Canada

**Keywords:** oncology, molecular biology, immunology

## Abstract

To identify long non-coding RNAs (lncRNAs) involved in breast cancer immunosuppression, we analyzed lncRNA expression and their correlation with tumor-infiltrating lymphocytes, association with worse patient outcomes, and enrichment in breast cancer and identified LINC01929 was a top hit in breast and other cancer types. Knockdown of LINC01929 in breast cancer cell lines reduced cell survival, cell cycle progression, inhibited tumor growth, and altered the expression of genes involved in growth, immune, and antigen presentation pathways. LINC01929 acts as a competitive endogenous RNA, regulating a cancer-promoting and immunosuppressive microRNA-mRNA network. Targeting LINC01929 in breast cancer cells increases proteasomal activity, and cell surface levels of human leukocyte antigen calls I (HLA I) and programmed death-ligand 1 (PD-L1). In breast tumors, LINC01929 expression negatively correlated with T cell abundance. Targeting LINC01929 sensitized breast cancer cells to activated T cell killing. These findings identify LINC01929 as an immunotherapy target to overturn breast cancer immunosuppression.

## Highlights

• LINC01929 is an lncRNA that drives breast cancer cell immune evasion
• LINC01929 regulates a microRNA-mRNA network controlling antigen presentation
• Silencing LINC01929 boosts HLA I levels, proteasome activity, and PD-L1 levels
• Targeting LINC01929 sensitizes tumors to activated T cell-mediated killing

## Graphical abstract

**Figure:**
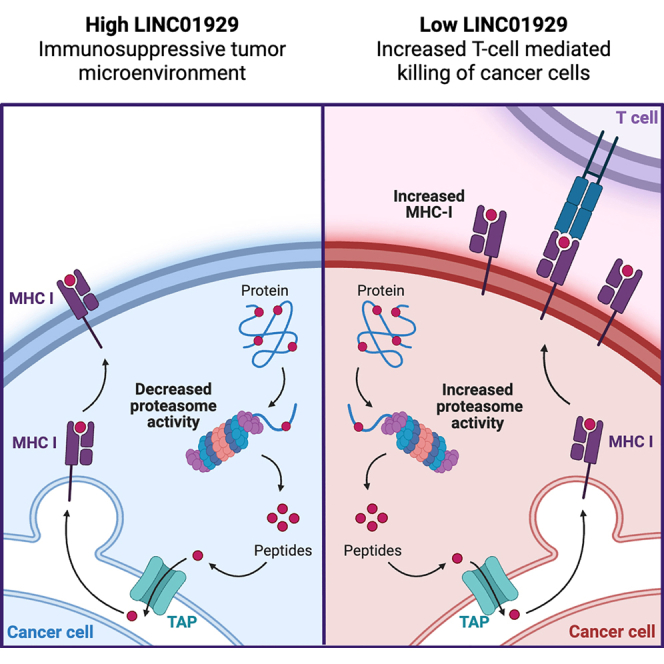

## Introduction

Cancer progression is influenced by the immune cells within the tumor microenvironment. An abundance of tumor infiltrating lymphocytes (TILs) is associated with improved cancer outcomes and greater efficacy of cancer therapies, particularly immune checkpoint inhibitors (ICIs).^1^ ICIs currently in the clinic enhance anti-tumor T cell activity by blocking interactions between the programmed cell death protein 1 (PD-1) receptor on T cells and its ligand PD-L1 on cancer cells and immunosuppressive leukocytes, or by blocking the cytotoxic T-lymphocyte-associated protein 4 (CTLA-4) on T cells.^2^ To successfully mediate these effects, ICIs require a “hot” tumor microenvironment: one that contains abundant cytotoxic T cells, supportive immune cells and cytokines, a high tumor mutational burden leading to increase presentation of neo-antigens, and the presence of PD-L1+ cells.^3^

ICIs are now used to treat a variety of cancers, including lung, melanoma, bladder, cervical, liver, stomach, and the aggressive triple-negative breast cancer (TNBC) subtype.^4^^,^^5^^,^^6^^,^^7^^,^^8^ While some patients benefit from ICIs, others do not; usually these non-responding patients harbor “cold” tumors, a feature common in non-TNBC breast cancers.^9^^,^^10^ Cold tumors lack sufficient cytotoxic CD8^+^ T cells, have immunosuppressive cells, and are characterized by low PD-L1 expression on cancer cells.^11^ Even tumors with a more immune-active microenvironment (“hot” tumors) may not respond to ICI when immune evasion and suppressive mechanisms outweigh local anti-tumor immunity.^11^ These mechanisms include reduced expression of major histocompatibility complex class I (MHC I, human leukocyte antigen [HLA] in humans) and inefficient production and presentation of peptides from neoantigens or tumor-associated antigens.^11^ Identifying targetable regulators of immune resistance mechanisms could inform the development of more effective immunotherapies. For breast cancer, targeting these factors could increase the efficacy of ICIs and extend their use to “cold” tumors, such as hormone receptor positive and human epidermal growth factor receptor 2 overexpressing (HER2+) subtypes.

The non-coding transcriptome is a largely untapped resource for therapy targets. Cancer progression, including its interaction with the immune system, is influenced by changes in the transcriptional landscape of cells, with non-coding RNAs playing a pivotal role in regulating gene expression.^12^ Among these, long non-coding RNAs (lncRNAs) are particularly notable. Despite their abundance, which is comparable in number to protein-coding genes, lncRNAs are understudied. LncRNA transcripts are longer than 200 nucleotides in length, lack protein coding potential, and can significantly influence cellular outcomes.^13^^,^^14^ Through sequence complementarity and secondary structures, lncRNAs interact with RNA, chromatin and protein, and can function as competitive endogenous RNA (ceRNAs, i.e., “sponges”) of microRNAs (miRNAs)^15^^,^^16^ to modulate gene expression and cell signaling pathways. This versatility positions lncRNAs as key players in cancer biology and potential therapeutic targets.

While genetic screens have been used to identify functional lncRNAs in specific contexts, these screens typically rely on cell lines, which may not reflect clinically relevant conditions.^17^^,^^18^^,^^19^ Alternatively, many lncRNA discoveries have been made through analysis of data from The Cancer Genome Atlas (TCGA), focusing on expression patterns in tumors and their correlations with clinical features.^20^^,^^21^^,^^22^^,^^23^ Lack of conservation of many human lncRNAs with the mouse genome hinders the use of syngeneic mouse models for identifying immunosuppressive human lncRNAs.^24^ In this study, we leveraged TCGA data to prioritize candidate lncRNAs for functional investigation by identifying those negatively correlated with TILs, enriched in tumor tissue, and association with worse patient survival. We identified 25 lncRNAs with these characteristics in breast cancer, with LINC01929 emerging as a leading candidate when a pan-cancer analysis was incorporated. Subsequent functional studies demonstrated that LINC01929 is highly immunosuppressive in breast cancer. Targeting LINC01929 holds promise for enhancing immunotherapy efficacy, as its inhibition promotes the features needed to overcome ICI resistance, including increased PD-L1 and HLA I expression, and T cell killing.

## Results

### Pan-cancer analysis identifies top lncRNAs negatively correlated with TILs, enriched in tumors, and associated with poor patient survival in breast and other cancers

To identify candidate lncRNAs for study with potential tumor immunosuppressive function in breast cancer that could be therapeutic targets, we leveraged the expression data for 12,727 lncRNAs from The Atlas of Non-coding RNA in Cancer (TANRIC).^25^ This lncRNA expression data originates from TCGA and hence can be paired with the TCGA patient tumor clinical data (i.e., survival) and the TIL estimates that have been completed for TCGA cohorts by histopathology analysis of the digitized hematoxylin and eosin (H&E)-stained images.^26^

**Figure 1:**
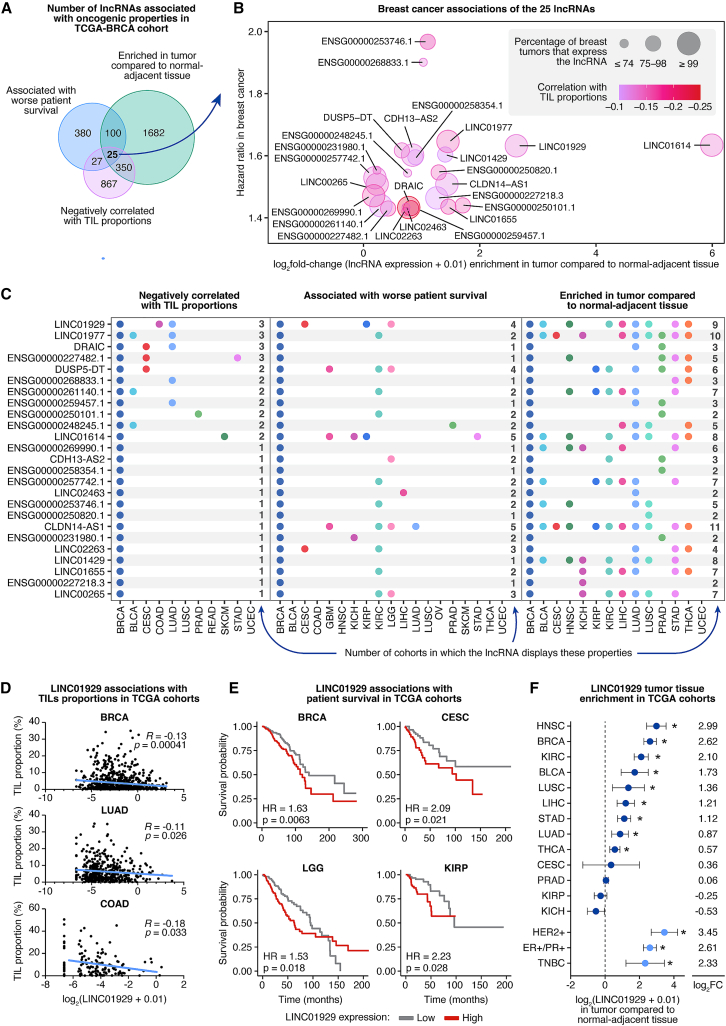
LINC01929 is a potential therapeutic target for breast and other cancers (A) Associations between lncRNA expression (RPKM RNA-seq) and tumor-infiltrating lymphocyte (TIL) proportions, patient survival, and tumor tissue enrichment in breast cancer were analyzed using the TCGA-BRCA and corresponding TANRIC datasets. LncRNAs that were negatively correlated with TIL proportions (Pearson coefficient <0, *p* value <0.05, *n* = 763), associated with worse patient survival (hazard ratio [HR] > 1, *p* value <0.05, *n* = 837), and enriched in tumor tissue compared to adjacent normal tissue (log_2_FC > 0, adjusted *p* value <0.05, *n* = 837 tumor samples, *n* = 105 normal-adjacent samples) were identified. (B) The percentage of breast tumors in the TCGA-BRCA dataset expressing each lncRNA (RPKM >0, represented by dot size) is displayed, along with their Pearson correlations with TIL proportions (dot color), hazard ratios (*y* axis), and tumor enrichment (*x* axis) for the 25 lncRNAs exhibiting the three characteristics analyzed in (a). (C) The associations with the 25 lncRNAs identified in breast cancer were further explored across all available TCGA datasets (listed on the *x* axis). Dots indicate TCGA cohorts where the lncRNA is negatively correlated with TIL proportions (left), associated with worst patient survival (center), or enriched in tumor compared to normal-adjacent tissue (right) are indicated with a dot. The total number of cohorts with significant associations for each lncRNA is quantified on the right side of each section. TCGA cohorts where LINC01929 shows significant Pearson correlations (*R*) with TIL proportions (D), associations with worst patient survival (E) are displayed. (F) The log_2_ fold change in lncRNA expression (RPKM RNA-seq) between tumor and normal-adjacent tissue is shown for the 13 TANRIC datasets with normal-adjacent samples, as well as for the ER+/PR+, HER2+, and TNBC breast cancer subtypes from the TCGA-BRCA dataset. Error bars represent 95% confidence intervals, and ∗ indicates an adjusted *p* value <0.05.

We first focused our analysis specifically on the breast cancer cohort (breast adenocarcinoma, TCGA-BRCA) and identified lncRNAs that met three criteria: (1) a negative correlation with TILs, (2) a correlation with poor patient survival, and (3) tumor tissue enrichment. This identified 25 lncRNAs (Figure 1A, Supplemental file S1).

Although most of the 25 lncRNAs are functionally unstudied, a few, such as LINC01614, lncRNA downregulated in cancer (DRAIC), and LINC00265,^27^^,^^28^^,^^29^^,^^30^^,^^31^^,^^32^ have been well-investigated and have been shown to promote the progression of cancer. Multiple studies have shown that LINC01614 promotes breast cancer progression.^33^^,^^34^^,^^35^ There is currently no evidence that any of the lncRNAs functionally suppress immune cell interactions in the tumor microenvironment; however, DRAIC has been associated with tumor immunosuppression phenotypes in multiple cancers (including breast cancer),^36^^,^^37^^,^^38^^,^^39^^,^^40^ and LINC01614, LINC00265, LINC01929, and LINC01655 expression has been associated with immune cell infiltration.^41^^,^^42^^,^^43^^,^^44^^,^^45^

We next compared the magnitudes of the correlations of the 25 lncRNA in breast cancers (Figure 1B). Among the 25 lncRNAs, DRAIC, ENSG00000259457.1, LINC02263, and ENSG00000269990.1, have the strongest negative correlations with TILs in breast cancer (Figure 1B). The lncRNAs have varied expressions in breast tumors, with 11 of the 25 lncRNAs expressed in over 99% of breast cancers (including LINC01614, LINC00265, DRAIC, LINC01929, and LINC01977). Among the 25 lncRNAs, LINC01614 and LINC01929 are the most highly enriched in breast tumors over normal-adjacent tissues. ENSG00000253746.1, ENSG00000268833.1, LINC01977, LINC01614, and LINC01929 have the highest hazard ratios (i.e., strongest associations with worse breast cancer patient outcomes, Figure 1B).

The 25 lncRNAs are of interest for further analysis; however, to prioritize which lncRNA to study, we expanded our analysis to include all TCGA and TANRIC cohorts. We reasoned that lncRNAs with negative prognostic correlations across multiple cancer cohorts, rather than just breast cancer, are more likely to have functional significance and may also be relevant targets for other cancers. Among the 25 lncRNAs, LINC01929, LINC01977, DRAIC, and ENSG00000227482.1 had negative correlations with TIL abundance in three cancers including breast cancer (Figure 1C, left). Four of the lncRNAs (LINC01929, DUSP5-DT, claudin 14-antisense 1 (CLDN14-AS1), and LINC01614) were associated with worse patient survival (significant hazard ratios greater than 1) in at least four of the TCGA cohorts (Figure 1C, center). Finally, three lncRNAs (LINC01929, LINC01977, and CLDN14-AS1), were in enriched tumor tissue compared to adjacent normal tissue in at least nine of the TCGA cohorts (Figure 1C right).

The breast cancer specific analysis (Figure 1B) combined with the pan-cancer analysis (Figure 1C) highlighted LINC01929 as a top lncRNA of interest for further study. LINC01929 is negatively correlated with TIL frequency in breast cancer (BRCA), lung adenocarcinoma (LUAD), and colon adenocarcinoma (COAD) (Figure 1D). Additionally, LINC01929 is associated with worse survival outcomes in breast cancer, cervical squamous cell carcinoma (CESC), low-grade glioma (LGG) and kidney renal papillary cell carcinoma (KIRP) (Figure 1E). Furthermore, its expression is enriched in the tumor tissue of nine TCGA cohorts (Figure 1F). LINC01929 is enriched all three clinical breast cancer subtypes (Figure 1F) as well as in male breast cancers (Figure S1). Interestingly, we noted that several transcription factors have been validated to bind to the promoter region of LINC01929 (Figure S2A). Among them, correlation analysis revealed that transcription factor SPI1 (which is upregulated in breast cancer^46^), is enriched in breast tumor versus normal adjacent tissues from the TCGA-BRCA cohort (like LINC01929) and is correlated with the expression of LINC01929 (Figures S2B and S2C). A recent pan-cancer TCGA analysis from another group also identified LINC01929 as highly enriched in 10 different cancer types and associated with worse survival in bladder, breast, colorectal, kidney and liver cancers.^47^ Accessing exoRBase 3.0 (a repository mRNA, lncRNA, and circRNA in extracellular vesicles from human biofluids),^48^ revealed that LINC01929 is enriched in the extracellular vesicles of blood samples of melanoma and gastric cancer patients (Figure S3), suggesting some liquid biopsy biomarker potential for these cancers. Given the strong overall correlations with clinically relevant characteristics, we prioritized LINC01929 for functional assessment.

### Targeting LINC01929 decreases breast cancer cell proliferation, survival, cell cycle progression, mammosphere formation, and tumor growth

**Figure 2:**
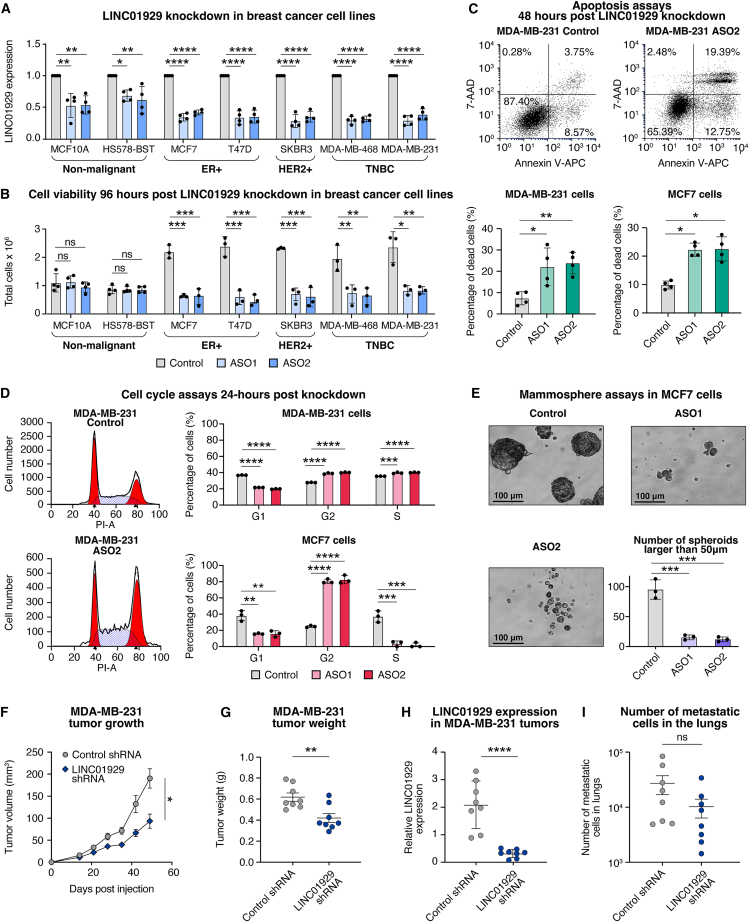
Targeting LINC01929 inhibits breast cancer cell proliferation, survival, cell cycle progression, mammosphere formation, and reduces tumor growth (A) qPCR analysis of LINC01929 expression 48 h after knockdown with control or LINC01929-specific antisense oligonucleotides (ASO1 and ASO2) in non-malignant and breast cancer cells (*n* = 4). Statistical significance was assessed using a one-way ANOVA followed by Dunnett’s multiple comparisons test. Error bars represent the standard deviation. (B) Total cell numbers were quantified 96 h post-LINC01929 knockdown in a panel of non-malignant (*n* = 4) and breast cancer cells (*n* = 3). Statistical significance was determined using a one-way ANOVA followed by Dunnett’s multiple comparisons test. Error bars represent the standard deviation. (C) Representative images (top) and quantification (bottom) of apoptotic cells 48 h after LINC01929 knockdown in MCF7 and MDA-MB-231 cells (*n* = 4), assessed by flow cytometry. Statistical significance was determined using a one-way ANOVA followed by Dunnett’s multiple comparisons test. Error bars represent the standard deviation. (D) Representative images (left) and quantification (right) of cell cycle assays in MCF7 and MDA-MB-231 cells following LINC01929 knockdown (*n* = 3). Statistical significance was assessed using a one-way ANOVA followed by Dunnett’s multiple comparisons test. Error bars represent the standard deviation. (E) Representative images and quantification of mammosphere formation assays in MCF7 cells following LINC01929 knockdown (*n* = 3). Scale bars of length 100 μm are depicted in the figure. Statistical significance was determined using a one-way ANOVA followed by Dunnett’s multiple comparisons test. Error bars represent the standard deviation. (F) Mean tumor growth of MDA-MB-231 tumors in mice treated with control shRNA (*n* = 8) or LINC01929 shRNA (*n* = 8). Statistical significance was determined by a paired *t* test. Error bars represent the SEM. (G) Tumor weights of MDA-MB-231 tumors in mice treated with control shRNA (*n* = 8) or LINC01929 shRNA (*n* = 8). Statistical significance was determined by an unpaired *t* test. Error bars represent the SEM. (H) qPCR validation of LINC01929 expression in control and knockdown MDA-MB-231 tumors. Statistical significance was determined by an unpaired *t* test. Error bars represent the standard deviation. (I) Quantification of metastatic cells in the lungs of mice bearing MDA-MB-231 tumors with or without LINC01929 knockdown. Statistical significance was determined by an unpaired *t* test. Error bars represent the SEM. *p* values are indicated as follows: ∗*p* < 0.05, ∗∗*p* < 0.01, ∗∗∗*p* < 0.001, ∗∗∗∗*p* < 0.0001 and ns = not significant.

LINC01929 is located on chromosome 18 and encodes 11 transcripts, ranging from 400 to 3,067 bases in length (Figure S4). To evaluate the functional role of LINC01929 in breast cancer, we generated antisense oligonucleotide (ASO) to specifically target the LINC01929 transcripts (Table S1). Treatment of normal and breast cancer cell lines with LINC01929-specific ASOs versus a negative control ASO showed a marked reduction of LINC01929 after 48 h (Figure 2A). This led to a reduction of cell numbers 96 h later in a panel of breast cancer cell lines representing the three breast cancer subtypes (with repeated ASO application at 48 h, Figure 2B). The reduction in cell numbers caused by LINC01929 knockdown was specific to breast cancer cells, as no notable changes were observed in the two non-malignant breast epithelial cell lines, MCF-10A and HS578-Bst.

The decreased cell numbers observed 96 h post ASO treatment suggests that LINC01929 affects cell survival and/or cell proliferation. For further analysis, we selected the hormone receptor positive MCF7 and TNBC MDA-MB-231 cells. We evaluated the percentage of apoptotic cells with annexin V and 7-AAD staining 48 h post-knockdown and noted an increase in dead cell frequency (Figure 2C). Additionally, LINC01929 knockdown impeded cell cycle progression, with a reduction in the proportion of cells in G1 and an accumulation in G2 (Figure 2D), indicating G2/M cell-cycle arrest.^49^

LINC01929 knockdown reduced mammosphere formation (Figure 2E), suggesting that inhibiting LINC01929 in breast cancer impairs the tumor-forming capacity/stemness properties of cancer cells.^50^ We further examined the *in vivo* effects of LINC01929 knockdown by mammary fat pad implantation of MDA-MB-231 cells stably expressing control shRNA or LINC01929 shRNA into non-obese diabetic/severe combined immunodeficiency (NOD/SCID) female mice. LINC01929 knockdown reduced tumor volumes and final tumor weights (Figures 2F and 2G). Analysis of extracted RNA from harvested tumors confirmed reduced LINC01929 levels in the tumors bearing the shRNA against LINC01929 (Figure 2H). We quantified metastatic MDA-MB-231 cells in the lungs and found no insignificant effects on metastasis (Figure 2I). Together these results indicate that LINC01929 exerts breast cancer-specific effects on cell survival, cell proliferation and promotes breast tumor growth, consistent with another recent study on this lncRNA.^45^

### LINC01929 alters the expression of genes involved in antigen presentation, stem cell differentiation, immune and T cell activation, protein catabolism, apoptosis, cell growth, and cell cycle processes

**Figure 3:**
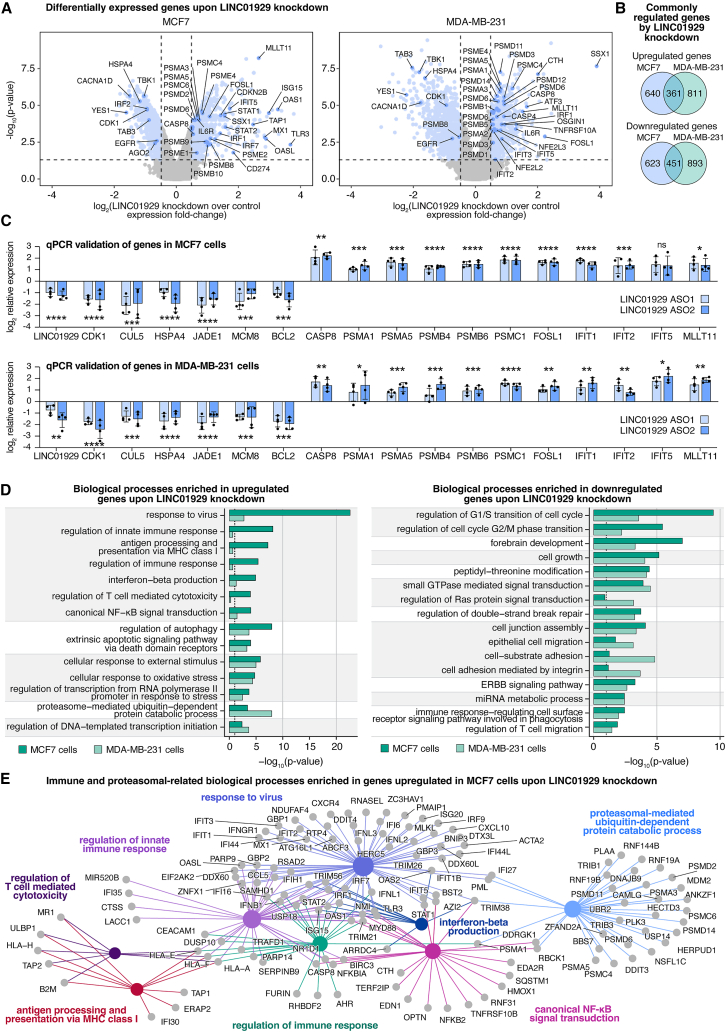
LINC01929 regulates genes involved in apoptosis, migration, cell cycle, and immune processes in breast cancer cell lines (A) Log_2_ fold changes in gene expression following LINC01929 knockdown compared to control in MCF7 (left) and MDA-MB-231 (right) cells, as determined by microarray (*n* = 3). A significance threshold of *p* < 0.05 and fold-change cutoffs of ±1.2311 were applied. Labeled genes represent those of particular interest. (B) The number of upregulated and downregulated genes meeting the specified cutoffs in both MCF7 and MDA-MB-231 cells were quantified. Overlapping upregulated and downregulated genes between the two cell lines were identified. (C) qPCR validation of select differentially expressed genes from the gene array analysis in (a). Data represents relative expression in LINC01929 knockdown (ASO1 and ASO2) compared to ASO control treatments (*n* = 4). Statistical significance was determined using a one-way ANOVA followed by Dunnett’s multiple comparisons test. Error bars represent the standard deviation. (D) Gene ontology (GO) analysis of upregulated (left) and downregulated (right) genes meeting the significance thresholds in (a). Biological processes were grouped by common functional pathways. (E) Curated network of significant immune- and proteasome-related biological processes enriched in upregulated genes upon LINC01929 knockdown in MCF7 cells. *p* values are indicated as follows: ∗*p* < 0.05, ∗∗*p* < 0.01, ∗∗∗*p* < 0.001, ∗∗∗∗*p* < 0.0001, and ns = not significant.

To investigate the functional mechanisms of LINC01929 in the breast cancer cells, we conducted transcriptome analyses of MCF7 and MDA-MB-231 cells using the Affymetrix human gene 2.0ST gene array analysis, which provides the expression of 24,838 Entrez genes (Figure 3A, GSE277314). Among the genes upregulated by LINC01929 knockdown in both cell lines, we noted transcriptional regulators, such as AP-1 transcription factor subunit FOS like 1 (FOSL1) and a component of the super elongation complex, MLLT1, which increases RNA polymerase II activity. Additionally, immune response genes, including the interferon induced protein with tetratricopeptide repeats 1 (IFIT1), were upregulated. Conversely, downregulated genes included heat shock protein family A (Hsp70) member 4 (HSPA4), serine/threonine kinase TANK-binding kinase 1 (TBK1), and transcriptional repressor synovial sarcoma X breakpoint 1 (SSX1). Over a thousand genes were affected by LINC01929 knockdown (Figure 3B). Probability analysis based on the total number of genes in the array predicts less than 4% chance of random overlap between the two cell lines; therefore, the ∼40% overlap suggests common regulatory mechanisms by LINC01929. To confirm these findings, we performed real time quantitative polymerase chain reaction (qPCR) on a subset of the genes from cells treated with control or LINC01929 targeting-ASOs (Figure 3C). The qPCR results were consistent with the gene array data, showing common downregulation of the key cell cycle gene cyclin dependent kinase 1 (CDK1) and anti-apoptotic gene B cell lymphoma 2 (BCL2). We also observed upregulation of genes associated with extrinsic apoptosis and inflammation, including caspase 8 (CASP8), the IFITs, and proteasomal subunits (e.g., PSMs, Figure 3C).

The high overlap of LINC01929 regulated genes was further reflected in the gene ontology (GO) enrichment analysis of the upregulated and downregulated genes following LINC01929 knockdown in each cell line (Figure 3D, Supplemental file S2). Enriched GO terms include those related to apoptosis mediation, immune response regulation, and proteasomal subunits (Figure 3D). The most significantly enriched GO terms were among the upregulated genes and included antigen processing and presentation via MHC I, T cell receptor signaling pathway, activation of immune responses and hematopoietic stem cell differentiation (Figures 3D and 3E). The upregulation of GO terms associated with extrinsic apoptosis (Figure 3D, left) aligns with the increased apoptosis observed following LINC01929 knockdown (Figure 2C). The enriched GO terms for the downregulated genes included those related to cell growth and G1/S phase transition of the cell cycle (Figure 3D, right), which is consistent with the cell-cycle arrest observed following LINC01929 knockdown (Figure 2D).

The GO enrichment analysis highlighted that LINC01929 knockdown upregulates the ubiquitin proteasome system (Figures 3D and 3E). This could impact various cellular processes, including the cell cycle, apoptosis, transcriptional regulation, immune response and antigen presentation.^51^^,^^52^ Overall, the transcriptome analysis supports a potential role for LINC01929 in breast cancer progression and immunosuppression by inducing changes in gene expression.

### Cytoplasmic LINC01929 is a competitive endogenous RNA, decreases the expression of key miRNA biogenesis player, and plays a central role in a miRNA-mRNA cancer-promoting network

**Figure 4:**
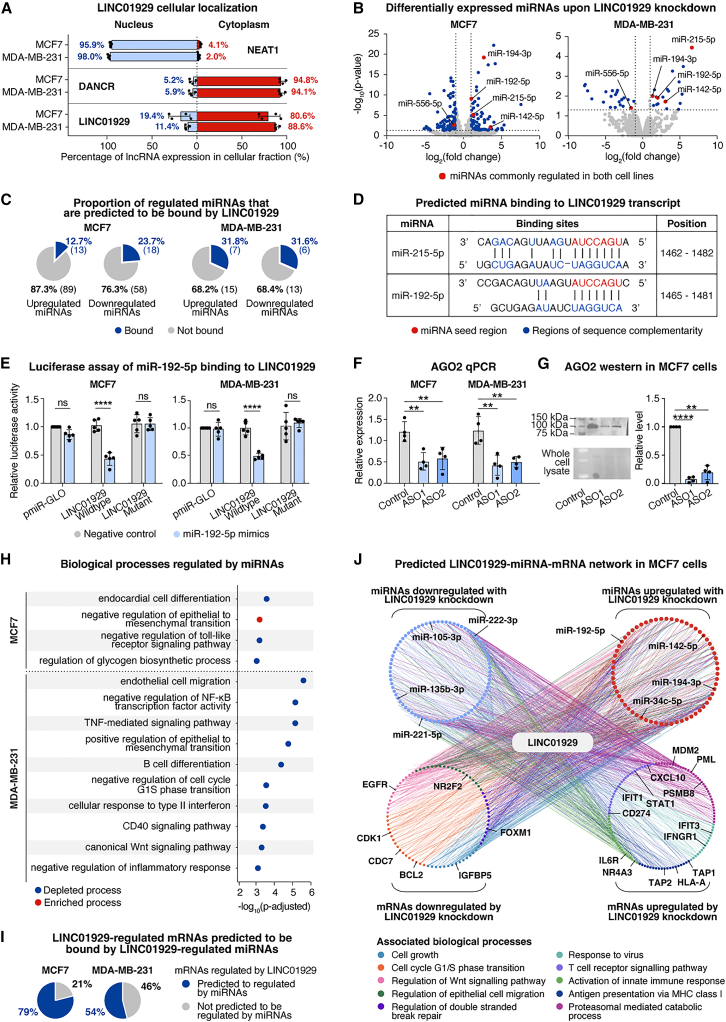
LINC01929 functions as a competitive endogenous RNA, regulating cancer-promoting pathways via a miRNA-mRNA network (A) The relative proportion of NEAT1, DANCR, and LINC01929 expression in nuclear and cytoplasmic fractions of MCF7 and MDA-MB-231 cells was measured by qPCR (*n* = 4). Error bars represent the SEM. (B) Log_2_ fold-change of miRNA levels in LINC01929 knockdown compared to control cells, determined by small RNA sequencing, in MCF7 (left) and MDA-MB-231 (right) cells (*n* = 3). A significance threshold of *p* < 0.05 and fold-change of ±1 was applied. The upper quadrants display the number of significantly upregulated or downregulated miRNAs. Red dots represent miRNAs significantly upregulated or downregulated in both cell lines. (C) Proportion of miRNAs meeting the criteria in (b) predicted to bind lncRNA, as determined by lncBook 2.0. (D) Predicted binding sites of miR-215-5p and miR-192-5p by lncbook 2.0, and their respective positions on the LINC01929 transcript. (E) Relative luciferase activity in MCF7 (left) and MDA-MB-231 (right) cells transfected with a pmirGLO dual-luciferase vector containing the predicted LINC01929 target sequence for miR-192-5p (wildtype) or a mutated version (mutant) and treated with miR-192-5p mimic or negative control (*n* = 5). Statistical significance was assessed by a two-way ANOVA followed by a Šídák multiple comparison test. Error bars represent the standard deviation. Luciferase activity was normalized to cells treated with the negative control mimic and wildtype sequence vector. (F) qPCR analysis of AGO2 expression in MCF7 and MDA-MB-231 cells after LINC01929 knockdown (*n* = 4). Statistical significance was determined by a one-way ANOVA followed by Dunnett’s multiple comparison test. Error bars represent the SEM. (G) Western blot analysis (left) and quantification (right) of AGO2 protein levels in control and LINC01929 knockdown conditions in MCF7 cells (*n* = 4). Statistical significance was determined by a one-way ANOVA followed by Dunnett’s multiple comparisons test. Error bars represent the SEM. (H) Gene ontology (GO) analysis showing biological processes enriched (red dots) or depleted (blue dots) in miRNAs regulated by LINC01929, meeting the criteria in (b), in MCF7 (top) and MDA-MB-231 (bottom) cells. (I) Predicted target mRNAs of miRNAs identified in (b) were cross-referenced with mRNAs regulated by LINC01929 knockdown, as shown in Figure 3A, using MultiMiR. The proportions of LINC01929-regulated mRNAs predicted to be modulated by miRNAs altered upon LINC01929 knockdown are shown. (J) Curated network of predicted LINC01929-miRNA-mRNA interactions in MCF7 cells, displaying mRNAs and miRNAs involved in cancer-promoting processes that are regulated by LINC01929 knockdown. *p* values are indicated as follows: ∗*p* < 0.05, ∗∗*p* < 0.01, ∗∗∗*p* < 0.001, ∗∗∗∗*p* < 0.0001 and ns = not significant.

Given the prominent gene expression changes observed with LINC01929 knockdown in breast cancer cells, we next investigated potential mechanisms of LINC01929 gene regulation. The cellular localization of lncRNAs can provide insights into their function. Cytoplasmic lncRNAs often regulate gene expression post-transcriptionally by interacting with miRNAs, while nuclear-localized lncRNAs typically interact with chromatin.^53^ To determine the localization of LINC01929, we fractionated MCF7 and MDA-MB-231 cell lysates into nuclear and cytoplasmic fractions. Our analysis revealed that LINC01929 was predominantly cytoplasmic in the breast cancer cell lines, like the well-characterized cytoplasmic lncRNA DANCR (Figure 4A).

The cytoplasmic location of LINC01929 suggests it may regulate miRNAs in breast cancer cells, aligning with prior LINC01929 studies in other cancers. In oral squamous cancer, LINC01929 “sponged” miR-137-3p, leading to upregulation of FOXC1.^54^ In bladder cancer, LINC01929 “sponged” miR-6875-5p, leading to increased ADAMTS12,^55^ while in non-small cell lung cancer, LINC01929 interacted with miR-1179.^56^ To investigate whether LINC01929 functions as a miRNA sequester in breast cancer, we performed small RNA sequencing on MCF7 and MDA-MB-231 cells (Figure 4B, GSE277592). The knockdown of LINC01929 resulted in the downregulation of 102 miRNAs and upregulation of 76 miRNAs in MCF7 cells, and the downregulation of 22 miRNAs and upregulation of 19 miRNAs in MDA-MB-231 cells. Among these, four miRNAs were commonly upregulated (i.e., miR-192-5p, miR-194-3p, miR-142-5p, and miR-215-5p), and miR-556-5p was commonly downregulated across both cell lines. Notably, all the commonly upregulated miRNAs have been reported to exhibit tumor suppressive functions in multiple cancers,^57^^,^^58^^,^^59^^,^^60^ aligning with the reduced cancer-promoting effects observed with LINC01929 knockdown. Among these, miR-192-5p and miR-215-5p belong to the miR-192 family of microRNAs and have been implicated in numerous diseases.^61^ In breast cancer, miR-192-5p was shown to increase breast cancer cell sensitivity to doxorubicin by targeting peptidylprolyl isomerase A (PPIA).^62^

The most common reported mechanisms by which lncRNAs regulate miRNA gene expression is through acting as a ceRNA or “sponge”, where the lncRNAs binds to the miRNAs via sequence complementarity.^53^^,^^63^ This interaction leads to the degradation/blocking of miRNAs, preventing them from binding to their mRNA targets.^64^^,^^65^ Consequently, some of the miRNAs which are upregulated upon LINC01929 knockdown may be “sponged” by LINC01929. This suggests that LINC01929 may be acting as a ceRNA to regulate the expression of multiple miRNAs.

We cross-referenced miRNAs regulated by LINC01929 with those predicted to bind LINC01929 using the lncbook 2.0 tool.^13^ Consistent with the hypothesis that reduced LINC01929 results in the upregulation of miRNAs through “sponging”, the percentage of miRNAs predicted to bind LINC01929 was highest among the upregulated miRNAs (Figure 4C). We found that the commonly upregulated miRNAs miR-192-5p and miR-215-5p share a binding site for LINC01929 in their seed region (Figure 4D). To validate this interaction between LINC01929 and miR-192-5p, we performed a miRNA luciferase reporter assay.^66^^,^^67^ A significant decrease in luciferase activity was observed only with the wildtype target sequence following treatment with the miR-192-5p mimic, confirming the interaction between LINC01929 and miR-192-5p in both MCF7 and MDA-MB-231 cells (Figure 4E). A similar assay in MCF7 cells confirmed the interaction of LINC01929 with miR-215-5p (Figure S5).

The changes in miRNA levels induced by LINC01929 cannot be fully explained by the predicted binding analysis alone, suggesting additional mechanisms of miRNA regulation. Our transcriptomic analysis suggested that LINC01929 knockdown results in the downregulation of miRNA metabolic processes (enriched GO term, Figure 3D, right), including key miRNA biogenesis gene argonaut 2 (AGO2, Figure 3A), which is required for the maturation of precursor-miRNAs.^68^^,^^69^ We confirmed that LINC01929 knockdown reduces AGO2 transcript levels by qPCR (Figure 4F) and detected a corresponding reduction in AGO2 protein level by western blotting in MCF7 cells (Figure 4G, all replicate blots shown in Figure S6). This decrease in AGO2 may contribute to decreased levels of mature miRNAs observed following LINC01929 knockdown (Figure 4B). Hence, the impact of LINC01929 on miRNAs appears to be multi-faceted and involves both ceRNA effects and alterations in miRNA biogenesis.

To evaluate the potential functional consequences of the miRNAs regulated by LINC01929, we performed gene set enrichment analysis (GSEA) and overrepresentation analysis using the miEAA V2.1 tool^70^ (Figure 4H). The GSEA revealed that the differentially expressed miRNAs are involved in several cancer-promoting pathways like those regulated by mRNAs altered by LINC01929 knockdown, such as immune processes and cell cycle regulation (Figure 3D, Supplemental file S3). This suggests that LINC01929 orchestrates a common regulatory network impacting both miRNAs and mRNAs.

We used the MultiMiR tool^71^ to identify potential mRNA targets among the genes regulated by LINC01929 (Figure 3A) and the corresponding downregulated and upregulated miRNAs (Figure 4B). In MCF7 cells, 95% of the mRNAs regulated by LINC01929 are predicted targets of the miRNAs altered by the lncRNA (Figure 4I). Similarly, in MDA-MB-231 cells, 63% of the regulated mRNAs are predicted targets of the regulated miRNAs (Figure 4I). These findings suggest that most gene expression changes induced by LINC01929 may be attributed to its effects on miRNAs. The resulting miRNA-mRNA networks were refined to highlight selected interactions enriched in GO processes (Figures 4J and S7), positioning LINC01929 at the center of a miRNA-mRNA network that mediates cancer-promoting biological processes in breast cancer. To further investigate gene regulation by LINC01929 (Figure 3C) through miRNAs, we analyzed the expression of selected genes upon treatment with miR-215-5p and miR-192-5p (Figure S8). Treatment of MCF7 and MDA-MB-231 cells with both mimics downregulated AGO2, confirming the LINC01929-miR-192-5p-AGO2 and LINC01929-miR-215-5p-AGO axes. However, the selected immune genes regulated by LINC01929 were not differentially expressed upon treatment of the mimics suggesting alternate mechanisms or possible regulation through many of the other regulated miRNAs.

### Targeting LINC01929 increases proteasomal activity, and cell surface HLA I and PD-L1 levels

**Figure 5:**
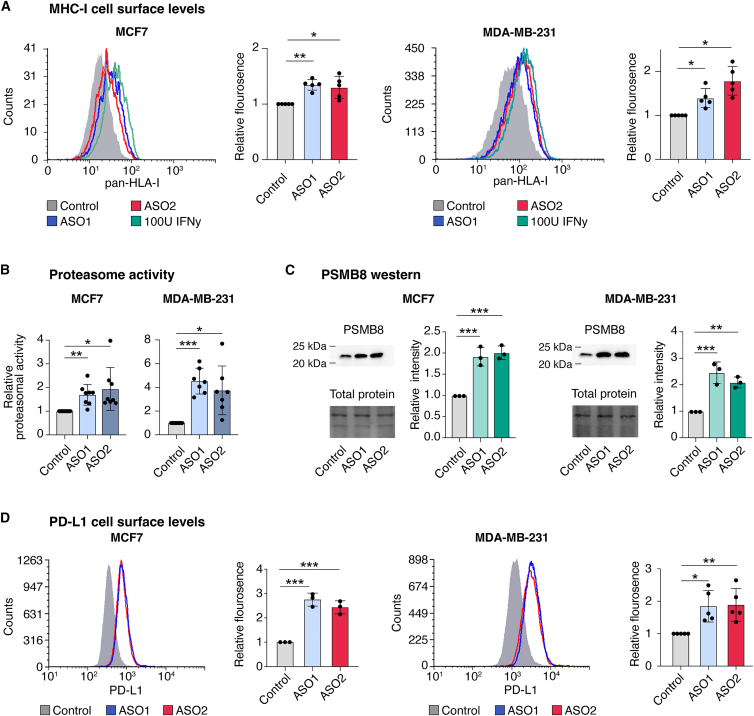
LINC01929 knockdown increases proteasome activity, and cell surface HLA I and PD-L1 (A) Representative images (left) and relative fluorescence intensity of cell surface HLA I (MHC I) levels in MCF7 and MDA-MB-231 cells following LINC01929 knockdown (*n* = 5). Significance was determined by a one-way ANOVA followed by Bonferroni multiple comparisons test. Error bars represent the standard deviation. (B) Relative proteasomal activity in MCF7 and MDA-MB-231 cells after LINC01929 knockdown (*n* = 7). Significance was determined by a one-way ANOVA followed by Dunnett’s multiple comparisons test. Error bars represent the standard deviation. (C) Western blot analysis of PSMB8 levels in MCF7 and MDA-MB-231 cells and quantification (*n* = 3). Error bars represent the standard deviation. (D) Representative images (left) and relative fluorescence intensity of cell surface PD-L1 levels in MCF7 (*n* = 3) and MDA-MB-231 (*n* = 5) cells following LINC01929 knockdown. Significance was determined by a one-way ANOVA followed by Dunnett’s multiple comparisons test. Error bars represent the standard deviation. *p* values are indicated as follows: ∗*p* < 0.05, ∗∗*p* < 0.01, ∗∗∗*p* < 0.001, ∗∗∗∗*p* < 0.0001.

The transcriptome data suggests that LINC01929 acts as an immune regulator in breast cancer, influencing the expression of genes enriched in pathways related to antigen presentation and immune cell activation (Figures 3 and 4). We assessed two key elements of antigen presentation machinery, cell surface expression of MHC I/HLA I and proteasome activity. The proteasome generates peptides compatible with MHC I, stabilizes MHC I on the cell surface, contributes to “hot” tumors, and is associated with favorable patient outcomes.^72^ Notably, our transcriptome analysis revealed increased expression of the components of the ubiquitin proteasome system in the LINC01929 knockdown cells (Figure 3). Flow cytometry analysis showed that LINC01929 knockdown increased MHC I (i.e., HLA I) on both MCF7 and MDA-MB-231 cells (Figure 5A). Additionally, proteasome activity increased in the cells following LINC01929 knockdown (Figure 5B). Although constitutively expressed proteasomes contribute to MHC I peptide generation, the immunoproteasome is specialized for this function.^72^ Our transcriptome data revealed increased expression of immunoproteasome-specific subunit genes PSMB8, 9 and 10 in MCF7 cells (Figure 3A). A western blot confirmed increased PSMB8 (proteasome subunit beta-5i) protein levels in MCF7 and MDA-MB-231 cells (Figure 5C, uncropped blots shown in Figure S9). The observed increase in proteasome activity and cell surface MHC I levels following LINC01929 knockdown is expected to enhance T cells targeting of the cancer cells.

High PD-L1 expression is characteristic of “hot” tumors; PD-L1 expression in these tumors is often induced by IFN-γ secreted by infiltrating activated T cells as an adaptive immune resistance mechanism.^11^^,^^73^^,^^74^ PD-L1 often increases the efficacy of anti-PD-1 ICIs, which depend on elevated PD-L1 levels on cancer cells to promote anti-tumor immune responses.^75^ Our transcriptome analysis suggests that, at least in MCF7 cells, LINC01929 knockdown leads to increased expression of CD274, the gene encoding PD-L1 (Figure 3A). Flow cytometry further confirmed that PD-L1 cell surface levels were elevated in both MCF7, and MDA-MB-231 cells 48 h post LINC01929 knockdown (Figure 5D). Collectively, these findings suggest that targeting LINC01929 shifts breast cancer cells toward a “hot” tumor phenotype and inhibits the immune evasion strategies common in cancer cells, elevated cell surface MHC I/HLA I and PD-L1, and enhanced proteasome activity.

### LINC01929 expression is negatively correlated with T cell infiltration in breast tumors and targeting LINC01929 increases killing of breast cancer cells by activated T cells

**Figure 6:**
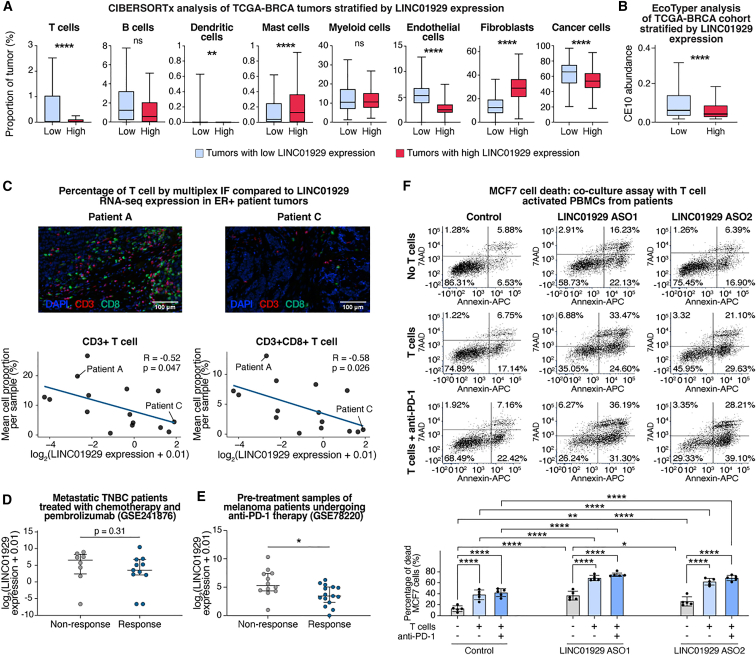
LINC01929 negatively correlates with T cell infiltration and immunotherapy response in breast tumors, and its knockdown enhances T cell-mediated killing of breast cancer cells (A) Tumor cell type proportions were estimated using CIBERSORTx in TCGA-BRCA tumors categorized by high (*n* = 261) and low (*n* = 247) LINC01929 expression levels, based on the top and bottom 33rd percentiles. Statistical significance was evaluated using the Mann-Whitney test, with box and whisker plots generated using the Tukey method. Error bars represent the standard deviation. (B) CE10 immune phenotype abundance was analyzed via EcoTyper in TCGA-BRCA tumors, comparing high (*n* = 421) versus low (*n* = 421) LINC01929 expression groups. Statistical significance was determined by the Mann-Whitney test, and box and whisker plots were created using the Tukey method. (C) Multiplex immunofluorescence staining (DAPI, CD3^+^, and CD8^+^) was performed on 15 hormone receptor positive breast tumor samples, with LINC01929 expression quantified by RNA sequencing. Representative images are shown for Patient A (low LINC01929 expression) and Patient C (high LINC01929 expression) (top). Pearson correlation coefficients (R) between CD3^+^ and CD3^+^CD8^+^ T cell proportions and LINC01929 expression are presented (bottom). Scale bars of length 100 μm are depicted in the figure. (D) LINC01929 expression was measured in metastatic triple-negative breast cancer (TNBC) patients treated with chemotherapy and pembrolizumab, comparing responders (*n* = 11) and non-responders (*n* = 8). Statistical significance was assessed using the Mann-Whitney test, with medians and interquartile ranges shown. (E) Pre-treatment LINC01929 expression levels were evaluated in melanoma patients treated with anti-PD-1 therapy, comparing responders (*n* = 15) and non-responders (*n* = 12). Statistical significance was determined by an unpaired *t* test, with medians and interquartile ranges displayed. (F) PBMCs from five different breast cancer patients, which had undergone CD3 CD28 T cell activation, were co-cultured with MCF7 cells (3:1 ratio of PBMCs to MCF7 cells) treated with either LINC01929-targeting or control ASOs, in the presence or absence of anti-PD-1. Representative images (top) and quantification of MCF7 cell death percentages (bottom, *n* = 5) are provided. Statistical significance was determined using two-way ANOVA with Tukey’s multiple comparisons test. Error bars represent standard deviation. *p* values are represented as: ∗*p* < 0.05, ∗∗*p* < 0.01, ∗∗∗*p* < 0.001, ∗∗∗∗*p* < 0.0001 and ns = not significant.

The initial selection of LINC01929 for study was partly based on its negative expression correlation with TIL abundance in breast cancer and other TCGA cohorts (Figure 1). While this analysis provides an estimate of the percentage of immune cells in TCGA tumors, it does not differentiate between TIL subsets. To further define the proportion of specific cell types, we stratified TCGA-BRCA tumors based on low or high LINC01929 expression and applied the deconvolution algorithm CIBERSORTx^76^ to the RNA-seq data. This revealed that LINC01929 expression is negatively correlated with the estimated frequencies of T cells, dendritic cells, endothelial cells, and cancer cells, and is positively correlated with mast cells and fibroblasts (Figure 6A). These findings suggest that LINC01929 is associated with “cold” immunosuppressive tumor microenvironment, as T cells and dendritic cells are crucial for tumor control and immunotherapy responses,^77^ while mast cells and fibroblasts promote immunotherapy resistance.^78^^,^^79^ Further EcoTyper analysis^80^ on the TCGA-BRCA RNA-seq data revealed that low LINC01929 expression is most associated with the carcinoma ecotype 10 (CE10, Figure 6B), which correlates with improved overall survival, increased immune cell filtration, active IFN-γ signaling, and a favorable response to immunotherapy.^80^

To investigate the relationship between LINC01929 expression and immune cell populations suggested by the deconvolution algorithms (Figures 6A and 6B), we quantified LINC01929 expression using RNA-seq and assessed immune cell populations by Opal multiplex immunofluorescence staining in an independent cohort of 15 local hormone receptor positive breast cancer patients. The inclusion of only hormone receptor positive tumor samples eliminated subtype-specific effects in this small cohort. Our analysis revealed negative correlations between LINC01929 expression and both total CD3^+^ T cells and the CD8^+^ T cell cytotoxic subset, (Figure 6C, Supplemental file S4). We also profiled B cells (CD20), pan-leukocytes (CD45), and cancer cells (panCK) and noted a nonsignificant negative trend between LINC01929 levels and B cell percentages in the tumor samples (Figure S10).

The negative association of LINC01929 with T cell infiltration in breast tumors (Figures 6A–6C), combined with our gene expression and phenotypic profiling of breast cancer cells (Figures 3 and 5), suggests that high LINC01929 expression may serve as a predictor of ICI resistance. We assessed published RNA-seq datasets of pre-treatment breast cancers patients treated with ICI therapy. Datasets with LINC01929 expression data are limited, but we were able to examine a cohort of 17 pre-treatment metastatic TNBC patients treated with carboplatin, nab-paclitaxel, and anti-PD-1 pembrolizumab (GSE241876).^81^ We noted a non-significant trend where responders (patients with complete and partial response) had lower average LINC01929 levels compared to non-responders (stable and progressive disease, Figure 6D). Additionally, in a cohort of 27 melanoma patients treated with anti-PD-1 therapies (pembrolizumab and nivolumab, GSE78220^82^), LINC01929 expression was lower in the responders compared to the non-responders (Figure 6E). These findings support the notion that LINC01929 could diminish the efficacy of ICI therapies.

Our data suggests that LINC01929 inhibition increases cell surface HLA I on breast cancer cells (Figure 5) and extrinsic apoptosis pathways (Figure 3), which could increase T cell-mediating killing, particularly as lower LINC01929 levels in breast tumors are associated with T cell infiltration (Figures 6A and 6B). To evaluate if LINC01929 inhibition increases the susceptibility of breast cancer cells to T cell killing, we performed co-culture assays using peripheral blood mononuclear (PBMC) with CD3/CD28-activated T cells from five different pre-treatment breast cancer patients (Figure S11 shows activation of the T cells). CD3/CD28 activation results in T cell killing that is not MHC I restricted,^83^ eliminating the need for antigen recognition in the assay. MCF7 cells, treated with control or LINC01929-targeting ASOs for 24 h, were then co-cultured with T cell activated PBMCs at a 1:3 ratio for an additional 24 h, with or without anti-PD-1 (Figure 6F). Flow cytometry analysis revealed increased T cell-mediated killing of the MCF7 cells treated with LINC01929-targeting ASOs (Figure 6F). Although not as effective as LINC01929 targeting alone, the inclusion of anti-PD-1 in the co-culture assays further increased the killing of breast cancer cells (Figure 6F). Together, these data suggest that LINC01929 inhibition could be a promising immunotherapy strategy for the treatment of breast cancer.

## Discussion

LncRNAs represent a largely untapped resource of potential therapeutic targets for the development of cancer treatments. Thousands of lncRNAs have been implicated in promoting cancer growth and metastasis^84^^,^^85^ and recent advances in antisense targeting platforms offer opportunities for therapeutic intervention.^86^ Identifying which of these cancer-promoting lncRNAs hold the greatest potential for clinical application remains a significant challenge. Given the increasing use of immunotherapy and the need to improve its efficacy in breast and other cancers, investigating lncRNAs with tumor immunosuppressive effects could lead to significant clinical advances. Several lncRNAs have already been shown to influence tumor immune responses. For instance, lncRNA capable of inducing MHC I and immunogenicity of tumor (LIMIT) upregulates MHC I and is positively correlated with TIL levels and ICI response in melanoma.^87^ Metastasis-associated lung adenocarcinoma transcript 1 (MALAT1) regulates PD-L1 expression in non-small cell lung cancers, while HOTAIR induces PD-L1 expression in laryngeal squamous cell carcinoma^88^ and glioma.^89^ MALAT1 was also shown to suppress immunogenic cell death and T cell killing of metastatic cancer cells.^90^ Epigenetically induced lincRNA 1 (EPIC1) suppresses IFN-γ cell signaling responses required and the expression of genes involved in antigen presentation required for T cell killing.^91^ LINC00973 sponges miRNAs targeting CD55 and CD59, leading to suppression of the complement system and CD8^+^ T cell activation, thus promoting tumor immune evasion.^92^ These examples underscore the emerging role of lncRNAs in modulating tumor immune responses, providing a strong foundation for their potential as therapeutic targets.

Here we identified LINC01929 as a master regulator of tumor immunosuppression and provide comprehensive analysis of its function and revealing its central role in a miRNA-mRNA network. To our knowledge, this is the first lncRNA found to downregulate multiple key components of the antigen presentation and processing pathway (i.e., HLA I cell surface levels and proteasome activity) in cancer cells. This discovery positions LINC01929 as a uniquely influential cancer-associated lncRNA, highlighting why its expression is elevated in many patient tumors compared to matched normal tissues; tumor cells likely depend on LINC01929 for survival. Our patient data analysis supports this cell line data, as breast tumors with high LINC01929 expression exhibit decreased CD8^+^ T cell infiltration and reduced IFN signaling, consistent with the phenotypes of “cold” tumors.

We considered investigating the effects of LINC01929 in breast tumors with T cells using syngeneic mouse tumor models with intact immune systems; however, challenges arose due to the lack of conservation of LINC01929 in mice, a common feature of lncRNAs.^93^ To address this, we utilized PBMCs isolated from breast cancer patient blood and performed co-culture assays with activated T cells that were not MHC I-restricted. This revealed that targeting LINC01929 in MCF7 cells increased T cell-mediated killing, surpassing the efficacy of anti-PD-1 therapy alone. Future studies that utilize overexpression experiments in both malignant and non-malignant cells could further delineate the mechanisms of LINC01929. However, it is important to note that lncRNAs often function within a complex regulatory network and may require additional malignant cues—such as altered transcriptional landscapes or tumor-specific signaling pathways—to exert immunomodulatory effects. Therefore, a lack of observable phenotype upon LINC01929 overexpression in non-malignant cells may not necessarily indicate biological irrelevance but rather suggest that LINC01929 alone is insufficient to induce immunosuppression outside a malignant context. Future studies examining the cooperative effects of LINC01929 and oncogenic pathways in transformation models may help clarify this aspect.

ICIs hold considerable promise and are increasingly used in the treatment of various cancers, including breast cancer.^2^ However, resistance to ICIs, particularly in “cold” tumors, remains a significant challenge, highlighting the need for strategies that can transform these tumors into a “hot” tumor microenvironment.^3^ Our study identified LINC01929 as a mediator of breast cancer progression and a key regulator of immunosuppressive gene expression. The potential to target LINC01929, combined with its enrichment in breast and other tumors, presents a promising strategy for counteracting immunosuppression and enhancing ICI. Although further research is needed to fully understand the function of LINC01929, our findings demonstrated its role in establishing a “cold” tumor microenvironment in breast cancers. Targeting LINC01929 could transform “cold” tumors into “hot” tumors, rendering cancer cells more susceptible to T cell-mediated killing. This approach holds substantial promise, especially for most breast cancers, which are frequently classified as “cold tumors” and exhibit limited response to ICI.^94^ By converting these tumors into a more immunologically active state, targeting LINC01929 could enhance the efficacy of ICI, or serve as an alternative strategy for ICI resistant tumors, such as those commonly seen in hormone receptor positive breast cancers.

### Limitations of the study

While this study provides extensive mechanistic evidence that LINC01929 is a central regulator of breast cancer immunosuppression, it should be noted that most functional experiments were performed in established 2D breast cancer cell lines, which do not fully recapitulate the cellular heterogeneity, immune architecture, or extracellular matrix interactions present in human tumors. Future studies using patient-derived organoids, 3D co-culture systems, or *ex vivo* tumor slice assays would provide more physiologically relevant validation.

## Resource availability

### Lead contact

Further information and requests for resources and reagents should be directed to and will be fulfilled by the lead contact, Paola Marcato (paola.marcato@dal.ca).

### Materials availability

This study did not generate new unique reagents.

### Data and code availability

• The datasets generated during the current study are available in the GEO repository, with the accession codes GSE277314 (gene array data) and GSE277592 (small-RNA sequencing data). Other transcriptome data used in this study were obtained from the publicly accessible TCGA and GEO databases as referenced in the corresponding sections.
• This article does not report original code.
• Any additional information required to reanalyze the data reported in this article is available from the lead contact upon request.

## Acknowledgments

The data presented in this paper were partially funded by the Terry Fox Research Institute’s Marathon of Hope Cancer Centers Network (MOHCCN). The small RNA sequencing analysis was carried out on high performance computing (HPC) clusters provided by the ACENET (https://ace-net.ca/) and the 10.13039/501100021202Digital Research Alliance of Canada (alliancecan.ca). The graphical abstract was designed with BioRender. The article was funded by grant support to P.M. from the 10.13039/501100000024Canadian Institutes of Health Research (10.13039/501100000024CIHR, PLL-185691 and PJT 190220). J.V. is a trainee of the Cancer Research Training Program (CRTP) of the 10.13039/100007669Beatrice Hunter Cancer Research Institute (BHCRI) and was supported by funds provided by GIVETOLIVE. M.-C.D. is funded by funds provided to P.M. from Canadian Breast Cancer Foundation-Atlantic Region Endowed Chair in Breast Cancer Research and donation made to Dalhousie University to support breast cancer research conducted by P.M.; M.-C.D. was funded by a doctoral award from the 10.13039/501100000024Canadian Institutes of Health Research (CIHR), a Killam Predoctoral Scholarship, Research Nova Scotia graduate studentship, and is a trainee of the CRTP BHCRI. R.P.A is funded by a I3V Dr. David H. Hubel Postdoctoral Fellowship, is a trainee of the CRTP-BHCRI and was funded by funds generously provided by the Canadian Cancer Society's JD Irving, Limited – Excellence in Cancer Research Fund. H.F.C. is funded by a Research Nova Scotia graduate studentship and a Nova Scotia Graduate Scholarship. O.L.W. is funded by a Killam Predoctoral Scholarship and the President's Award. W.F. was funded by the 10.13039/100007670Dalhousie Medical Research Foundation-I3V Dr. David H. Hubel Postdoctoral Fellowship. M.R.M is funded by Research Nova Scotia graduate studentship and a Dalhousie University Faculty of Medicine studentship. M.E.M was funded by a Faculty of Medicine Scholarship and a Nova Scotia Graduate Scholarship. B.K. is funded by a Killam Predoctoral Scholarship. P.S.K. is funded by the Donald Hill Family Postdoctoral Fellowship. S.N. and S.L are trainee members of the BHCRI. S.N. was supported by a Vanier Canada Graduate Scholarship from the 10.13039/501100000024CIHR and a Killam Predoctoral Award and Nova Scotia Graduate Scholarship through Dalhousie University. S.L is supported by a Canada Graduate Scholarship from the CIHR and a Nova Scotia Graduate Scholarship through Dalhousie University.

## Author contributions

J.V. and M.-C.D. conceptualized and designed the study and performed the experiments, data analysis, and data interpretation, and wrote the manuscript. R.P.A., H.F.C., O.L.W., C.A.D., W.F., M.R.M., M.E.M., B.K., P.S.K., M.S., S.N., S.L., M.N.L., C.S.H., and J.A.P. performed experiments and interpreted the data. D.G., T.J.B., G.C.K., G.B., S.G., J.E.B., and J.P.M. provided the materials for the study, interpreted data and/or performed analysis, supervised, provided guidance on study design, and reviewed and edited the manuscript. P.M. conceptualized, designed, and supervised the study, acquired funding, performed data interpretation and wrote the manuscript. All authors edited and revised the manuscript and were involved in the final approval of the manuscript.

## Declaration of interests

J.V., M.-C.D., and P.M. are co-founders of Oncolinc Therapeutics Inc., a startup aimed at developing targeted therapies against lncRNAs as immunotherapies. In addition, J.V., M.-C.D., R.P.A., H.F.C., B.K., and P.M. are authors on a US patent application titled “Methods and compositions targeting LINC01929 to increase antigen presentation” submitted in March 2025.

## STAR★Methods

### Key resources table

**Table:** 

REAGENT or RESOURCE	SOURCE	IDENTIFIER
**Antibodies**		

LMP7	Abcam	cat# ab180606 ; RRID:AB_3661893
AGO2	Cell Signaling	cat #2897; RRID:AB_2096291
PE anti-human HLA-A,B,C	Biolegend	cat# V 311405; RRID:AB_314874
InVivoMab anti-human MHC Class I (HLA-A, HLA-B, HLA-C)	Bio X Cell	Cat# BE0079, RRID:AB_1107730

**Bacterial and virus strains**		

DH5α	Fisher	Cat#: 18258012

**Biological samples**		

Fresh frozen- Breast cancer tumor samples	NSH	1023191
FFPE- Breast cancer	NSH	1028015

**Chemicals, peptides, and recombinant proteins**		

Recombinant IFN-γ	PeproTech	Cat # 300-02-100UG

**Critical commercial assays**		

RNeasy Mini Kit	Qiagen	74106
APC Annexin V Apoptosis Detection Kit	Biolegend	640930
Proteosome Activity Assay Kit	Abcam	ab107921
Opal 7-color manual IHC kit	Akoya Biosciences	**OP-000003**
iScript cDNA Synthesis Kit	Biorad	1708890
PARIS™ Kit	Thermofisher scientific	AM1921
mirVana™ miRNA Isolation Kit	Thermofisher scientific	AM1560
Pierce BCA Protein Assay Kit	Thermo fisher scientific	23225

**Deposited data**		

microarray data- LINC01929 KD	This paper	GSE277314
miRNA seq data- LINC01929 Knockdown	This paper	GSE277592
TCGA Cohort	Broad Genome Data Analysis Center	https://gdac.broadinstitute.org/
TIL- data for TCGA cohort	Saltz et al.	https://doi.org/10.7937/K9/TCIA.2018.Y75F9W1

**Experimental models: Cell lines**		

MCF7	ATCC	HTB-22; RRID:CVCL_0031
MDA-MB-231	ATCC	CRM-HTB-26 RRID:CVCL_0062
MDA-MB-468	ATCC	HTB-132 RRID:CVCL_0419
SKBR3	ATCC	HTB-30; RRID:CVCL_0033
T-47D	ATCC	HTB-133; RRID:CVCL_0553
Hs578Bst	ATCC	HTB-125 RRID:CVCL_0807
MCF10A	ATCC	RRID:CVCL_0598 CRL-10317

**Experimental models: Organisms/strains**		

NOD/SCID	Charles River Laboratories	

**Software and algorithms**		

bowtie2 software	Langmead and Salzberg	https://bowtie-bio.sourceforge.net/bowtie2/index.shtml
DeSeq2	Love et al.	https://bioconductor.org/packages/release/bioc/html/DESeq2.html
ggplot2	Wickham	https://ggplot2.tidyverse.org/
Graphpad Prism	Dotmatics	https://www.graphpad.com/
clusterProfiler R	Yu G et al.	https://bioconductor.org/packages/release/bioc/html/clusterProfiler.html
Limma	Ritchie ME et al.	https://bioconductor.org/packages/release/bioc/html/limma.html
FCS Express	DeNovo software	https://denovosoftware.com/
survminer v0.1.15	Kassambara	https://github.com/kassambara/survminer
MultimiR	Ru Et.al	http://multimir.org/
R Studio	The Comprehensive R Archive Network	https://cran.r-project.org/

### Experimental model and study participant details

#### Patient tumor sample and PBMC access

In all experiments, patient samples were obtained from individuals who had a clear understanding of their samples being accessed for experimental analysis and provided written consent for their samples to be submitted to the NSHA/Dalhousie Biobank. All study methodologies with patient tissues conformed to the standards set by the Declaration of Helsinki. The study methodologies were approved by the Nova Scotia Heath (NSH) and IWK Research Ethics Boards. The research ethics board (REB) numbers for the approved protocols are 1023191 (IWK REB, “Breast Biobanking”) and 1028015 (NSH REB, “Investigating genetic biomarkers of breast cancer progression and immunosuppression”). The treatment naïve tumor samples were taken at the time of the patients’ primary surgery and were preserved as fresh frozen samples (used for RNA isolation and RNAseq) and FFPE blocks (used for Opal multiplex immunofluorescence analysis).

For PBMC isolation, 5ml of patient blood was collected in a K2EDTA coated vacutainer™. Upon receiving, the blood was diluted 4-fold in PBS and layered on top of 15ml of lymphoprep™ (Stem cell technologies) without mixing the layers, on a sepmate ™ (stem cell technologies)50 ml centrifuge tube. The layered tube was centrifuged at 1200 X g for 20 minutes at room temperature with low break and acceleration for separating the PBMCs. The buffy coat containing the PBMCs was carefully siphoned off to a new tube, washed twice in PBS. The PBMCs were either directly used for T cell activation and co-culture assays or cryopreserved in CryoStor (Stem Cell Technologies, Vancouver, Canada).

#### Mouse models

Mouse experiments have been conducted in accordance with the ethical standards and according to the Declaration of Helsinki and the Canadian Council on Animal Care (CCAC) standards and were approved under animal protocol #21-011. Eight-week-old NOD/SCID female mice were injected with 2,000,000 MDA-MB-231 cells.

#### TCGA data access and other dataset access

LncRNA per kilobase of transcript per million mapped reads (RPKM) RNA-sequencing expression for 19 TCGA cohorts was accessed from TANRIC.^25^ A description of the TCGA studies utilized in this work, their corresponding abbreviations, and the number of tumors and normal-adjacent samples in each dataset are listed in Table S2. The corresponding clinical data for TCGA cohorts was accessed through the Broad Genome Data Analysis Center (GDAC) Firehoses (https://gdac.broadinstitute.org/). Additionally, the tumor-infiltrating lymphocyte (TIL) percentages of samples for 11 TCGA cohorts were previously quantified from the images of hematoxylin & eosin -stained slides and obtained from Saltz et al.^26^ We also accessed exoRBase 3.0: an atlas of mRNA, lncRNA and circRNA in extracellular vesicles from human biofluids to determine if LINC01929 was detected in these liquid biopsy samples.^48^

### Method details

#### Differential expression of TCGA datasets

The limma R package v3.54.2^95^ was utilized to identify differential expression of lncRNA transcripts between tumor and normal-adjacent samples within the 14 TCGA cohorts that contained normal-adjacent tumor samples (Table S2). Prior to analysis, the expression data was transformed to log2(expression + 0.01). To control false positive results, p-values were adjusted using the Benjamin-Hochberg (BH) correction. An adjusted p-value cutoff of 0.05 was used.

#### Survival analysis

Survival analyses were performed using the survival v3.2.13 R package,^96^ and the results were visualized using the survminer v0.1.5 R package.^97^ To conduct the analyses, patients were divided into “high” and “low” expression groups based on the median expression of each RNA transcript. The Cox proportional hazards model was then applied to determine the hazard ratio of each lncRNA across all breast cancer subtypes. LncRNA transcripts expressed in fewer than 15% of patients within a specific group were excluded from the analysis to ensure the result reliability. Additionally, to mitigate the impact of outliers, lncRNAs with hazard ratios below 0.05 or above 20 were removed from the analysis.

#### LncRNA expression correlations with tumor infiltration lymphocyte proportions

The Pearson correlation coefficient and corresponding p-values between lncRNA expression and TIL proportions from Saltz et al.^26^ were conducted the cor.test () function in R.

#### Cell culture

A description of cell lines, culturing conditions and media utilized in this study are found in Table S3.

#### Antisense oligonucleotide knockdown and cell proliferation assays

The transient knockdown of LINC01929 was achieved with screening-grade modified ASOs also called as locked nucleic acid (LNA) GapmeRs (Qiagen) which mediate RNase H–dependent degradation of target transcripts^98^^,^^99^ (sequences listed in Table S1). The ASOs/GapmeRs were designed using Qiagen GeneGlobe Antisense LNA Gapmer design portal. The negative control ASO, which does not bind anywhere in the genome (confirmed by a BLAST analysis), was also used as a corresponding control.^100^ ASOs were mixed with OptiMEM reduced serum media (Life Technologies, Thermo Fisher Scientific) and TransIT-BRCA transfection reagent (MJS Biolynk, Brockville, ON, Canada) and added to sub-confluent cells to a final treatment concentration of 15nM as per the manufacturer’s instructions. Knockdown was confirmed by quantitative polymerase chain reaction, at 48h post transfection as described below. The knockdown of LINC01929 to 96h was maintained by repeated transfection every 48 hours. Cell proliferation was determined by cell counting and trypan blue staining at 96 hours post initial transfection with the ASOs.

#### RNA extraction and real time quantitative PCR

For all transcript expression analyses by qPCR, cells were collected in TRIzol, and total RNA was purified using a PureLink RNA kit (Life Technologies, Thermo Fisher Scientific) as per the manufacturer’s instructions. Equal amounts of harvested RNA were reverse transcribed with iScript cDNA Synthesis Kit (Bio-Rad) as per the manufacturer’s instructions. QPCR was performed using SsoAdvanced Universal SYBR Super-mix (Bio-Rad) and transcript-specific primers (primer sequences are listed in Table S4) as per the manufacturer’s recommended protocol using a CFX96 Touch RealTime PCR Detection System (Bio-Rad). Primer efficiencies, determined by standard curves of diluted cDNA samples, were incorporated into the CFX Manager software (Bio-Rad). Gene expression for all samples was calculated relative to two or three reference genes and relative to the control ASO/GapmeR-treated samples.

#### Apoptosis assay

MCF7 and MDA-MB-231 cells were treated with 15nM negative control or LINC01929 ASOs for 48 hours. Cells were harvested by trypsinization with TrypLE Express (Life Technologies, Thermo Fisher Scientific) and washed with PBS. The cells were stained in Annexin V binding buffer with Annexin V and 7AAD using APC Annexin V Apoptosis Detection Kit with 7-AAD (Biolegend, San Diego, CA, USA) as per manufactures protocol. The cells were analyzed in BD FACSCelesta™ Multicolor Flow Cytometer (Becton Dickinson, Franklin Lakes, NJ, USA). The results were analyzed and plotted with FCS express (*De Novo* software, Pasadena, CA, USA).

#### Cell cycle assay

MCF7 and MDA-MB-231 cells were plated in 6 well plates at 30% confluency and cultured in serum deficient media to synchronize the cells. The synchronized cells were treated with 15nM negative control or LINC01929 ASOs for 24 hours. Cells were harvested by trypsinization with TrypLE Express (Life Technologies, Thermo Fisher Scientific) and washed with PBS. The cells were stained using FxCycle™ PI/RNase Staining Solution (Invitrogen, Thermo Fisher Scientific) as per manufacturer’s instructions. The cells were analyzed in BD FACSCelesta™ Multicolor Flow Cytometer (Becton Dickinson, Franklin Lakes, NJ, USA) and the results were plotted using Modfit LT (Verity Software House, Topsham, ME, USA).

#### Mammosphere assays

To assess the effects of LINC01929 knockdown on mammosphere formation potential, 1500 MCF7 cells were seeded in complete MammoCult media (Stemcell Technologies, Vancouver, BC, Canada) in technical triplicates in 24-well ultralow adherence plates (Thermo Fisher Scientific). Two hours post seeding, cells were treated with 15nM ASOs/GapmeRs as described above. All resulting spheres greater than 50μm (defined using the integrated software of an AE31E microscope (Motic, Richmond, BC, Canada), were counted 5 days later.

#### Mouse tumor studies

Mouse experiments have been conducted in accordance with the ethical standards and according to the Declaration of Helsinki and the Canadian Council on Animal Care (CCAC) standards and were approved under animal protocol #21-011. Eight-week-old NOD/SCID female mice were injected with 2,000,000 MDA-MB-231 cells stably expressing shRNA control or LINC01929-shRNA (sequences listed, Transomic (Now Skyang Bio), Huntsville, AL, USA, Table S5) cells admixed 1:1 with matrigel-HC (ThermoFisher Scientific, Waltham, MA, USA) into the mammary fat pad (n=8). Tumor volumes were quantified with caliper measurements (mm3, length × width × width/2). Final tumor weights were measured following termination and RNA was extracted from minced portions as described above to determine LINC01929 levels by qPCR. The lungs from the mice were harvested and the number of metastatic cells in the lungs were identified as previously described.^101^

#### Gene array

MCF7 and MDA-MB-231 cells were treated with LINC01929-specific or negative control ASOs for 48h and then collected in TRIzol reagent (n=3). RNA purification was performed as described above and sent to the Centre for Applied Genomics (TCAG, The Hospital for Sick Kids, Toronto, Canada) for Affymetrix Human Gene 2.0 ST gene chip platform analysis. The data were processed with the Transcriptome Analysis Console (Affymetrix) to reveal differential gene expression. The raw data and processed data files have been deposited at the Gene Expression Omnibus (GEO) and available at GSE277314.

#### Gene ontology enrichment

Genes that exhibited a fold-change threshold greater than 1.4 or less than -1.4, accompanied by a p-value below 0.05, were selected as input for Gene Ontology (GO) analysis. Enriched biological functions were identified using the clusterProfiler R package v4.6.2.^102^ BH correction was applied to obtain adjusted p-values. Processes with a p-value < 0.05 and q-value < 0.2 were investigated.

#### Cellular fractionation

Fractionation of the cells were done using PARIS™ Kit (ThermoFisher scientific) as per manufacturers instruction. Briefly, MCF7 and MDA-MB-231 cells were collected and lysed in cold cell fractionation buffer, to separate the cytosolic and nuclear fractions. The nuclear pellet was further lysed in cell disruption buffer. The RNA from both the nuclear and cytoplasmic fractions was extracted as described above. LINC01929 levels were determined by qPCR and compared to nuclear NEAT1^103^ and cytoplasmic DANCR.^104^

#### Small RNA-sequencing and differential expression of miRNAs

Small RNA was extracted from MCF7 and MDA-MB-231 cells with or without LINC01929 knockdown with GapmeRs with mirVana™ miRNA Isolation Kit (Invitrogen, Thermo Fisher Scientific) as per manufacturer’s instructions. Total RNA from same lysates was used to confirm knockdown of LINC01929 using qPCR as described. Samples were sent to TCAG for Illumina NovaSeq - SP flowcell. The quality of the raw data was verified with FastQC^105^ and Cutadapt V 4.0^106^ was used to trim NEBNext Small RNA library adaptors and retained reads between 12-51 nucleotides in length. rRNA reads were removed with SortmeRNA Release v4.3.7.^107^ Bowtie2 v2.5.1^108^ was used to align reads to the reference genome (hg38 assembly). Samtools^109^ was used to align and index files. FeatureCount V 2.0.1^110^ was used to annotate the .bam file based on annotation from the miRbase. edgeR (v 4.0)^110^ was used to identify differentially expressed miRNAs across LINC01929 knockdown and control conditions. The data files are deposited on GEO and accessed at GSE277592. The gene set enrichment analysis for the miRNAs was performed using miEAA V 2.0.^70^ The mRNA binding of differentially expressed miRNAs was predicted using MultiMir. The resulting LINC01929/miRNA/mRNA network was visualized using Cytoscape V 3.2.^111^

#### Luciferase reporter assay for LINC01929-miR-192-5p interaction

Oligos specific to the wildtype (WT) LINC01929-miR-192-5p binding region and the mutated version of the sequence (MUT) are listed in Table S6. To make double stranded sequences for cloning, the oligos were admixed into oligo annealing buffer and heated to 90 °C for 3 minutes, followed by cooling to 37 °C for 15 minutes. The WT and MUT annealed oligos (ThermoFisher Scientific) were cloned into the multiple cloning site of the pmirGLO Dual-Luciferase miRNA Target Expression Vector (ThermoFisher Scientific, using SacI and XhoI restriction enzymes (New England Biolabs Ltd.). The confirmed vectors were co-transfected into MCF7 and MDA-MB-231 cells with the pRLTK vector (Promega ThermoFisher Scientific), using TransIT-BRCA transfection reagent. 24 hours later the mirVana miRNA negative control mimic or mimic-hsa-miR-192-5p (ThermoFisher Scientific) was transfected into the cells using TransIT-BRCA. The resulting firefly and renilla luciferase activity in the cells were measured 24 hours later using the Dual-Glo® Luciferase Assay System (ThermoFisher Scientific) with a SpectraMax® M3 Multi-Mode Microplate Reader (ThermoFisher Scientific). Binding the mimic sequence to the luciferase reporter vector would inhibit production of luminescence.

#### Luciferase reporter assay for LINC01929-miR-215-5p interaction

The oligos designed for miR-192-5p also contain the binding region for miR-215-5p as well and the assay was performed as described above in MCF7 cells. After transfection of the plasmids, the cells were treated with hsa-miR-215-5p miRCURY LNA miRNA Mimic (Qiagen, Gene globe ID: YM00470002-ADA) and Negative Control 5 miRCURY LNA miRNA Mimic (Qiagen, Gene globe ID: YM00479904-ADA) and the luciferase activity was measured as described above.

#### MicroRNA-mimic assay

To identify the microRNA mediated transcriptional effects of the genes regulated by LINC01929 we treated MCF7 and MDA-MB-231 cells with miRNA mimics for miR-192-5p (Qiagen, GeneGlobe ID: YM00471355-ADA), miR-215-5p (Qiagen, Gene globe ID: YM00470002-ADA) and negative control mimic (Qiagen, Gene globe ID : YM00479904-ADA) for 24 hours. RNA was extracted and qPCR was performed for the gene panel as described above.

#### Western blotting

Cells were lysed in RIPA buffer and quantified with Pierce BCA Protein Assay Kit (Thermo fisher scientific). 50μg of the lysate was loaded in Mini-PROTEAN TGX Stain-Free Precast Gel (Bio-Rad) and ran for 1 hour at 100 V in Tris-Glycine-SDS buffer. The lysates were transferred onto PVDF membranes in a Transblot-Turbo Transfer system (Bio-Rad) and blocked in 5% milk in TBST for 1 hour at room temperature. The membranes were incubated with 1/1000 anti-PSMB8 (LMP7, cat# ab180606, Abcam, Cambridge, UK) or anti-AGO2 (cat #2897, Cell Signaling Technology, Danvers, MA, USA) diluted in 5% bovine serum albumin, overnight at 4 °C followed by peroxidase affiniPure goat anti-rabbit IgG (H + L, #111-035-144, Jackson Immunoresearch, West Grove, PA, USA) antibody (1:1000 in 5% milk TBST) for 1 hour at room temperature. The chemiluminescence was imaged with the ChemiDoc imaging system (Bio-Rad) and the band intensities were calculated and plotted using Imagelab software (Bio-Rad). Densitometric analysis on at least three biological replicates was performed using Image lab (Bio-Rad) and relative quantity was analyzed against the total protein. One-way ANOVA was performed for statistical analysis.

#### HLA I staining

MCF7 and MDA-MB-231 cells were treated with 15nM negative control or LINC01929 ASOs for 48h or 100-IU IFN-γ (PeproTech, Cranbury, NJ, USA). Cells were harvested by trypsinization with TrypLE Express (Life Technologies, Thermo Fisher Scientific) and washed with PBS. The cells were treated with PE anti-human pan HLA A, B,C antibody clone W6/32 (Biolegend), or PE Mouse IgG2a, κ Isotype Ctrl (MOPC-173) and 7-AAD for viability staining and incubated at room temperature for 30 minutes. All antibodies were obtained from Biolegend (San Diego, CA, USA) and concentrations were optimized for individual cell lines in preliminary assays. Cells were washed with PBS after incubation and resuspended in FACS buffer (PBS+1% EDTA+ 1-2% FBS) for analysis in BD FACSCelesta™ Multicolor Flow Cytometer (Becton Dickinson, Franklin Lakes, NJ, USA). The results were analyzed and plotted with FCS express (*De Novo* software, Pasadena, CA, USA).

#### PD-L1 staining

MCF7 and MDA-MB-231 cells were treated with 15nM negative control or LINC01929 ASOs for 48 hours. Cells were harvested by trypsinization with TrypLE Express (Life Technologies, Thermo Fisher Scientific) and washed with PBS. The cells were treated with APC anti-human CD274 (B7-H1, PD-L1) Clone 29E.2A3 (Cat #329708 Biolegend (San Diego, CA, USA)) as per manufacturer’s instructions. Cells were washed with PBS after incubation and resuspended in FACS buffer (PBS+1% EDTA+ 1-2% FBS) for analysis in BD FACSCelesta™ Multicolor Flow Cytometer (Becton Dickinson, Franklin Lakes, NJ, USA). The results were analyzed and plotted with FCS express (*De Novo* software, Pasadena, CA, USA).

#### Proteosome activity assay

The influence of LINC01929 on proteosome activity was studied using a fluorometric Proteosome Activity Assay Kit (Abcam, Cat# ab107921). MDA-MB-231 and MCF7 cells were seeded in 6 well plates treated with 15nM negative control or LINC01929 ASOs. 48 hours post knockdown, the GapmeR treated, and control cells were trypsinized, pelleted and lysed in ice cold 0.5% NP40. The proteosome activity was measured on the lysates using the AMC tagged fluorometric substrate, compared against standards, positive control and proteosome inhibitor against each sample, over two hours at 15 minutes intervals. The proteosome activity was calculated based on manufacturer’s protocol and normalized between samples for total protein using Bicinchoninic acid assay.

#### TCGA-BRCA RNA-seq deconvolution by CIBERSORTx

Cell proportions in the TCGA-BRCA cohort were estimated using the CIBERSORTx algorithm.^76^ A custom signature matrix was generated using a comprehensive breast cancer single cell RNA-sequencing (scRNA-seq) dataset,^112^ which included expression for 2697 B cells, 16235 T cells, 362 dendritic cells, 2119 endothelial cells, 5888 fibroblast cells, 363 mast cell, 1965 myeloid cells and 14395 cancer cells. Due to the size constraints of the CIBERSORTx website, a subset of 200 samples from each cell type was randomly selected using the seed () function in R to create a manageable file. The RPKM RNA-sequencing data from TCGA-BRCA tumors were deconvoluted using the custom signature matrix, applying B-mode batch correction and disabling quantile normalization, with 100 permutations. The estimated cell proportions of each tumor were then matched with the corresponding lncRNA expression data from TANRIC to assess the relationship between LINC01929 expression and tumor cell proportions.

#### TCGA-BRCA EcoTyper analysis

Cell state assignments for the TCGA-BRCA cohort were downloading the “TCGA Cell State Assignments” file from the Carcinoma EcoTyper section of the EcoTyper website. These assignments were matched with the corresponding lncRNA expression data from TANRIC. Tumors were then stratified into low and high LINC01929 expression groups based on the median expression, enabling the investigation of the relationship between cell states and LINC01929 expression.

#### RNA-seq of breast cancer patient tumor samples

15 hormone receptor positive breast cancer tumor fresh frozen samples were homogenized using a Qiagen TissueRuptor II for 30 seconds in buffer RLT Plus (with B-ME) and RNA extracted using the All Prep DNA/RNA Mini kit (Qiagen) as per manufacturer’s protocol. RIN values and DV200% were confirmed with Agilent TapeStation using RNA High Sensitivity. Library preparations were performed with Illumina Stranded Total RNA Prep with Ribo-Zero Plus as per manufacturer’s protocols and RNA libraries are run on the TapeStation to check the library size using the Agilent D1000 tapes.

Raw RNA sequencing data was processed using an analysis pipeline adapted from the Nextflow language-based workflow manager based nfcore pipelines.^113^^,^^114^^,^^115^ Briefly, raw FastQ files were preprocessed for initial QC and adaptors trimmed using fastp (version 0.23.4).^116^ Reads that met the analysis criteria were mapped to the human reference genome (GRCh38) using STAR (version 2.7.10a).^117^ BAMs were sorted and indexed using Samtools (version 1.16.1)^109^ and visualized using IGV (version 2.13.0).^118^ Duplicate reads were marked using Picard (version 3.1.0).^119^ Finally, the FeatureCounts (version 2.0.1),^120^ Salmon (version 1.10.1)^121^ and Tximport (Bioconductor -tximeta version 1.12.0 and r-base version 4.1.3)^122^ were used to perform transcript reconstruction and statistics including TPM count matrix generation. All software was used with default settings.

#### Opal multiplex immunofluorescence of breast cancer patient tumor fluorescent

The multiplex immunofluorescence protocol was adapted from Nersesian et al. 2023, and 2024. 5μm slides from the FFPE blocks of were de-paraffinized, rehydrated, and fixed in 10% neutral buffered formalin for 20 min. Antigen retrieval was conducted by microwave treatment (2 minutes at 100% power followed by 15 minutes at 20% power, 1000W microwave) in Tris-EDTA buffer (pH 9). Slides were cooled for 15 minutes at room temperature, then rinsed with deionized water, Tris-Buffered Saline, and Tween-20 (TBS-T) buffer. The tyramide signal amplification (TSA)-based IF staining protocol was conducted according to the Opal 7-color manual IHC kit. Slides were incubated in blocking buffer (Akoya Biosciences) for 10 minutes to stabilize epitopes and reduce background staining. Slides were then incubated with a primary antibody for 45 minutes, rinsed in TBST, and incubated with secondary anti-mouse and anti-rabbit HRP polymer for 10 minutes. Slides were rinsed and Fluorophore staining was then conducted with an Opal fluorophore in Opal amplification diluent for 10 minutes. The slides were then rinsed the protocol was sequentially repeated from the microwave treatment – to remove previous bound antibodies – to the Opal fluorophore. The steps were repeated for each antibody in the multiplex panels of CD8 (C8/144, prediluted, Dako/Agilent) and CD3 (LN10, 1/50, Monosan, Netherlands) or panCK (AE1/AE3, 1/50, Dako/Agilent), CD20 (L26, 1/25, Cell Signaling), CD3 (LN10, 1/50), CD45 (CD45-2B11, 1/50, ebioscience, Thermofisher Scientific). Following antibody and Opal staining, the slides were incubated with Spectral DAPI for 10 minutes, rinsed and mounted in Prolong Gold mounting media (Invitrogen, Thermofisher Scientific).

Multispectral imaging analysis was conducted following the protocol by Nersesian et al.,.^123^ Images were captured using a PhenoImager Quantitative Pathology workstation with multispectral separation capabilities, at 10 nm wavelength intervals from 420 nm to 740 nm, and 10x magnification. Images were visualized and processed in InForm Tissue Finder Software to conduct multispectral analysis (extracting fluorescent signatures) and subsequent quantitative pathology. Tissues were virtually segmented based on pan-cytokeratin staining to define regions such as tumor epithelia, tumor stroma, vasculature/autofluorescence, and off-core areas. This was followed by cellular segmentation to identify individual cell nuclei based on DAPI staining. Each cell was then assigned a unique cell ID which was used to quantify the antibody signal surrounding the individual nuclei. Antibody signals were used to phenotype the following cell types: T cells were identified as CD3^+^ or CD3+/CD45+ cells; B cells as CD20^+^ or CD45+/CD20+; leukocytes as CD45^+^; stromal cells as panCK-/CD45-; and cancer cells as panCK+/CD45-.

Analysis of the exported data was performed using the packages from Akoya Biosciences. Cell type proportions for each sample were calculated by Phenoptr and PhenoptrReports R dividing the number of cells of each type by the total cell count in the sample. The proportions for each cell type were then averaged to provide a single value per sample.

#### PBMC T cell activation and co-culture assays

CD8^+^ T cell activation and propagation from the patient PBMCs was performed using Human CD3/CD28 T cell Activation Beads (Biolegend), with slight modifications from the manufacturer’s protocol. The Isolated PBMCS were cultured in serum free immunocult ™-XF media (Stem Cell Technologies) for 24 hours prior to activation. The cells were collected and counted for viable cells and mixed with T cell activation beads at 1:1 ratio. The cells with beads were seeded at a seeding ratio of 600 X 103-750 X 103 cells/cm^2^. The T cell activation was supplemented with 10IU/ml of recombinant human IL2 (Peprotech) and 10ng/ml recombinant human IL15 (Biolegend) and 10% FBS (Gibco). After 72 hours of activation, the beads were removed by washing in PBS and cultured in the supplemented media for up to 10 days. The co-culture assays were performed from day 6 to day 10.

MCF7 cells were seeded at 100,000 cells /well in a 6 well plate and treated with 15nM negative control or LINC01929 ASOs 14 hours later. 24 hours post ASO treatment, the cancer cells are co-cultured with CD3/CD28 activated T cells at ratios of 1:3. The T cell supplement media was used for co-culture. 24 hours post co-culture, the culture media and the cells were harvested, centrifuged, washed, and stained with Annexin-V-APC/7AAD for viability staining. The percentage of live and dead cells were analyzed by flow cytometry (BD Celesta (BD)). Prior to co-culture the CD3/CD28 activated PBMCs were stained with CellTrace ™ CFSE (Thermo Fisher Scientific) to allow for identification of cancer cells and PBMCs in the flow cytometry analysis.

### Quantification and statistical analysis

All statistical analyses were performed in the GraphPad Prism software (GraphPad Software, San Diego, CA, USA), unless otherwise mentioned. In all cases where three or more groups are compared, a one-way or two-way ANOVA was performed (with Dunnett’s or Tukey’s multiple comparisons post-test as indicated in the figure legend). The corresponding figure legends describe the statistical test for each experiment, the number of data points (n) analyzed, what n represents, and the number of experimental replicates performed. All *in vitro* experiments were performed at least in triplicate, and the data are presented as the mean ± standard deviation (SD) as described in the figure legends. For animal experiments, the data are presented as the mean ± standard error of the mean (SEM) as described in the figure legend. Comparisons between two groups were done using a two-tailed student’s t-test. Significant p values are indicated as follows in the figures: p < 0.05 = ∗, p < 0.01 = ∗∗, p < 0.001 = ∗∗∗, p < 0.0001 = ∗∗∗∗, and ns = not significant.

## Supplemental information

Document S1. Figures S1–S11 and Tables S1–S6

Data S1. List of 25 candidate lncrnas meeting the selection criteria, related to Figure 1

Data S2. Gene ontology list of mRNAs regulated by LINC01929, related to Figure 3

Data S3. GSEA of miRNAs regulated by LINC01929, related to Figure 4

Data S4. LINC01929 association with CD3^+^ and CD8^+^ cells in 15 hormone receptor positive breast cancer patients, related to Figure 6
