# Supplementary material for: LINC01929 mediates breast cancer immunosuppression and is an immunotherapy target
Source: iScience. 2026 Mar 17;29(4):115381. doi: 10.1016/j.isci.2026.115381 (PMC13081058; doi:10.1016/j.isci.2026.115381)
Supplement: Document S1. Figures S1–S11 and Tables S1–S6 [file mmc1.pdf]

iScience, Volume *29*

Supplemental information

LINC01929 mediates breast cancer immunosuppression and is an immunotherapy target

Jaganathan Venkatesh, Marie-Claire D. Wasson, Raj Pranap Arun, Hannah F. Cahill, Olivia L. Walker, Cheryl A. Dean, Wasundara Fernando, Maya R. MacLean, Meghan E. McLean, Bakhmala Khan, Perryn S. Kruth, Mukhayyo Sultonova, Sarah Nersesian, Stacey N. Lee, Modeline N. Longjohn, Christopher S. Hughes, Thomas J. Belbin, Daniel Gaston, Joao A. Paulo, Gregory C. Knapp, Gillian Bethune, Shashi Gujar, Jeanette E. Boudreau, J. Patrick Murphy, and Paola Marcato

SUPPLEMENTAL FIGURES

Supplemental Figure S1.related to Fig.1

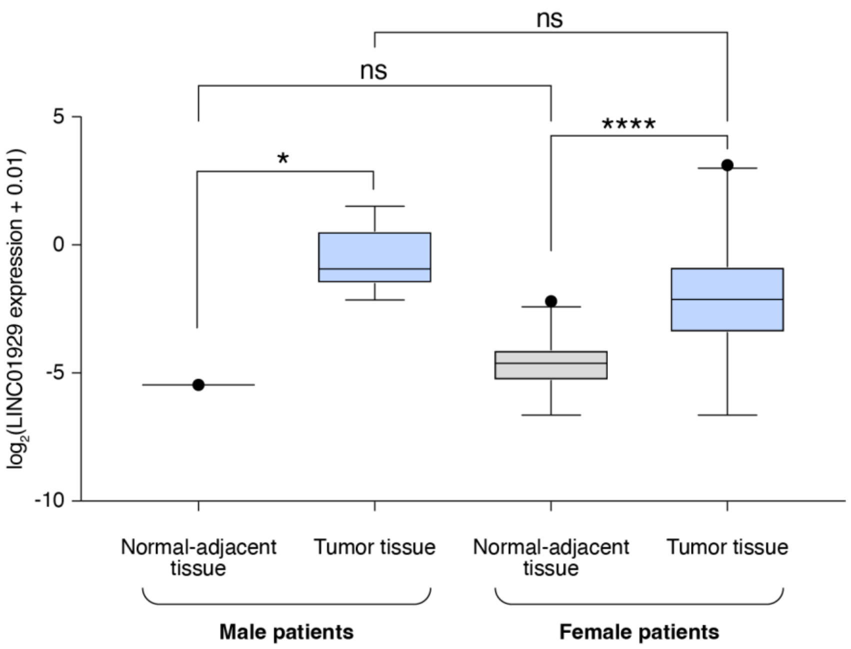

LINC01929 expression enrichment in male and female breast cancer patients versus normal-adjacent tissue. LINC01929 RPKM expression levels were derived from RNA-sequencing data of TCGA-BRCA patients using The Atlas for Non-Coding RNAs in Cancer (TANRIC), with the corresponding clinical data obtained from The Broad Institute Firehose. Patients were stratified by sex, including male (normal-adjacent: n = 1, tumor: n = 8) and female (normal-adjacent: n = 97, tumor: n = 706) cohorts. Boxplots depict the median and interquartile range, with whiskers determined by Tukey's method. Statistical analysis was performed using one-way ANOVA followed by Tukey's multiple comparisons test. p-values are denoted as: ns (not significant) = $p > 0.05$; * = $p < 0.05$; **** = $p < 0.0001$.

Supplemental Figure S2.related to Fig.1

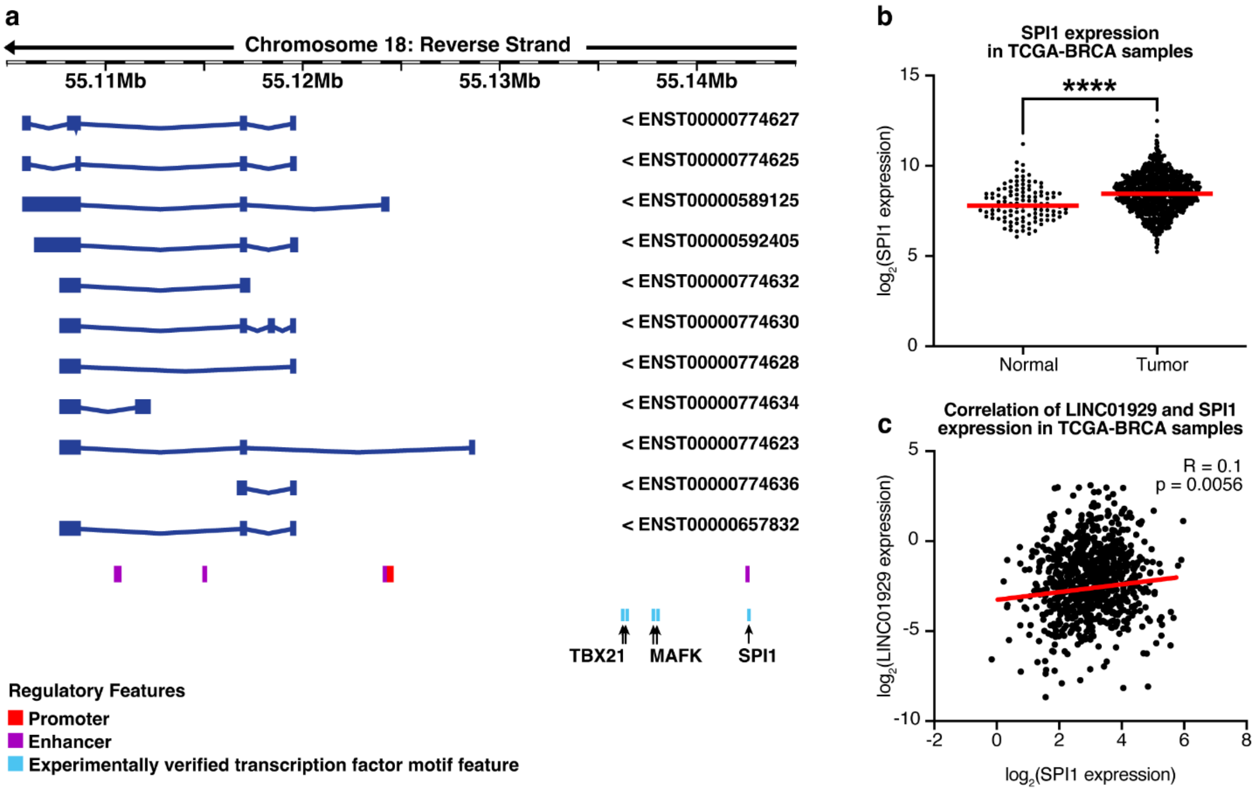

Transcription factors correlation with expression of LINC01929. (a) Transcription factors experimentally validated to bind within or upstream of LINC01929 gene were identified (data access from Ensembl). Among these, SPI1 is enriched in breast tumor compared to normal tissue samples in the TCGA-BRCA cohort **(b)** and is correlated with LINC01929 in the tumor samples from the same cohort, as assessed by Pearson correlation **(c)**.

Supplemental Figure S3.related to Fig.2

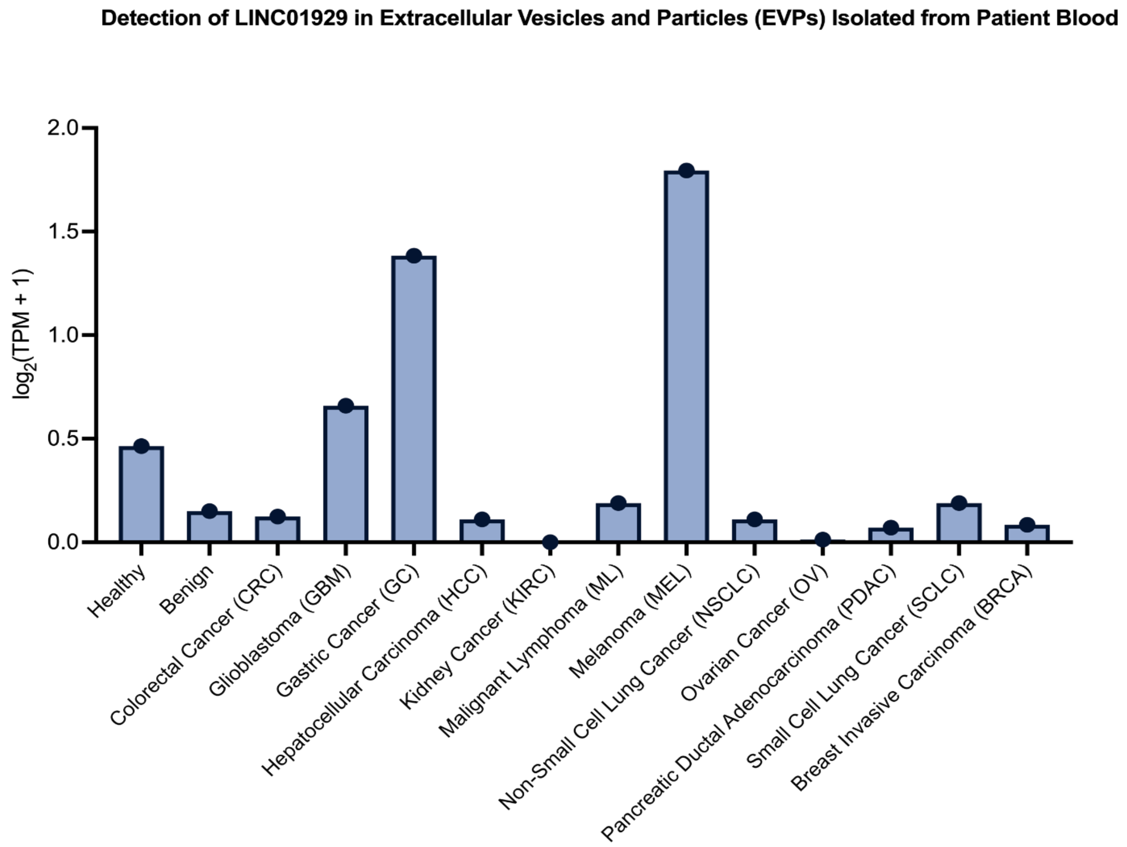

Detection of LINC01929 in extracellular vesicles and particles (EVPs) isolated from patient blood. Mean expression levels of LINC01929 are shown. Data were obtained from ExoRBase 3.0.

Supplemental Figure S4. related to Fig.3

Transcript	Length	Location on Chr18
ENST00000774627	752 bp	55,105,903-55,119,571
ENST00000774625	501 bp	55,105,903-55,119,603
ENST00000589125	3067 bp	55,105,904-55,124,306
ENST00000592405	2472 bp	55,106,499-55,119,682
ENST00000774632	1168 bp	55,107,786-55,117,253
ENST00000774630	1198 bp	55,107,786-55,119,563
ENST00000774628	960 bp	55,107,786-55,119,587
ENST00000774634	1438 bp	55,107,788-55,112,203
ENST00000774623	1081 bp	55,107,788-55,128,662
ENST00000657832	948 bp	55,107,788-55,119,584
ENST00000774636	400 bp	55,116,792-55,119,603

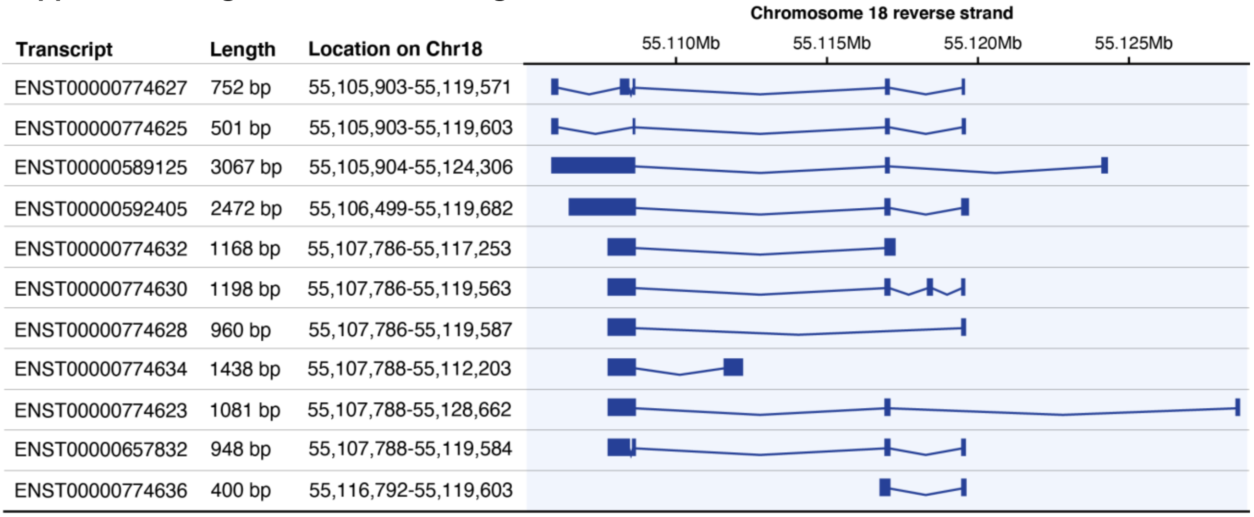

LINC01929 transcript variants. Transcript lengths and genomic positions for LINC01929 (ENSG00000267013) were retrieved from the Ensembl GRCh38.p14 human genome assembly.

Supplemental Figure S5.related to Fig.4

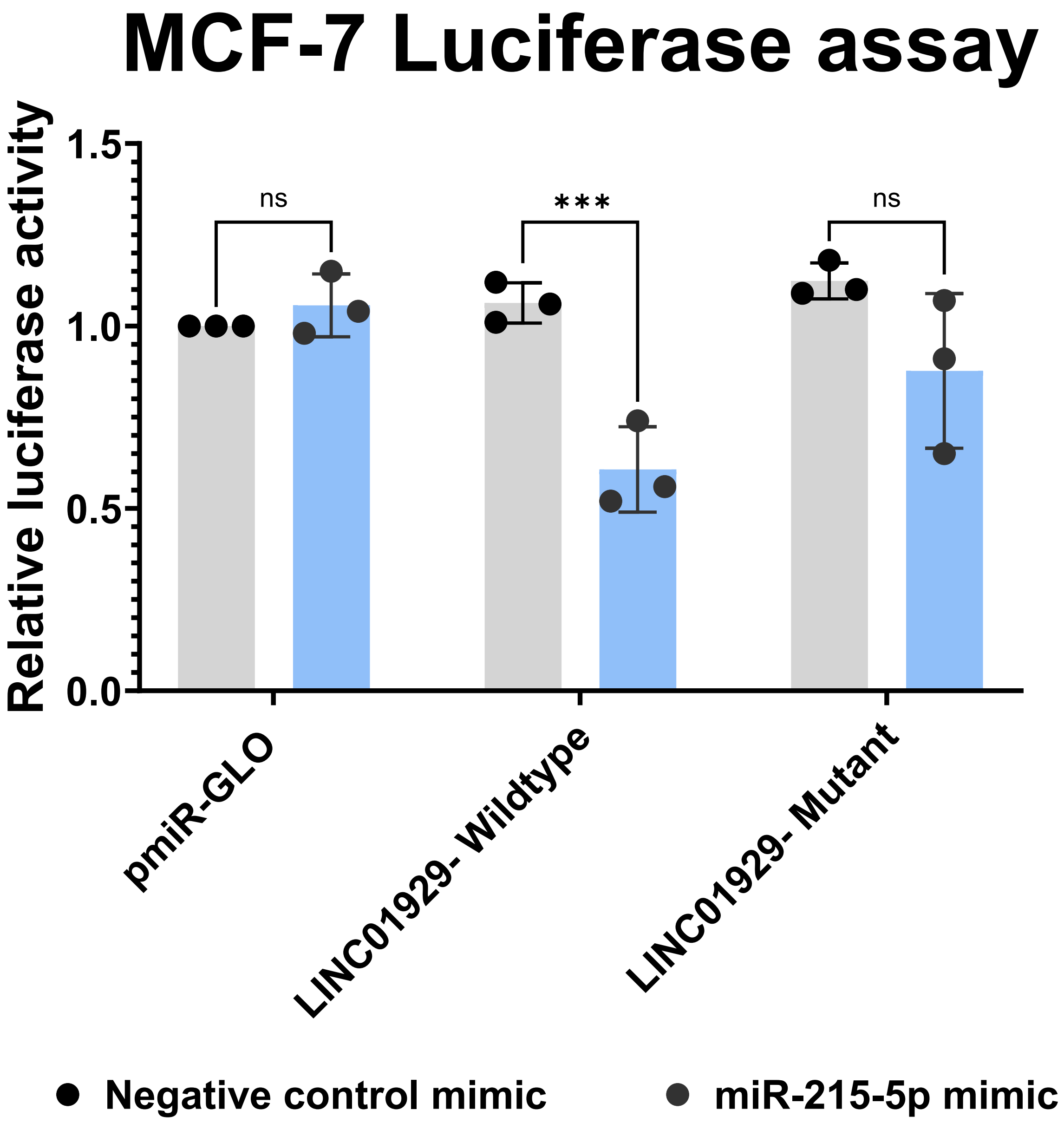

Luciferase assay of LINC01929 with miR-215-5p in MCF7 cells.

Relative luciferase activity in MCF7 cells transfected with a pmirGLO dual-luciferase vector containing the predicted LINC01929 target sequence for miR-192-5p (wildtype) or a mutated version (mutant) and treated with miR-215-5p mimic or negative control (N=3). Statistical significance was assessed by a two-way ANOVA followed by a Šídák multiple comparison test. Error bars represent the standard deviation. Luciferase activity was normalized to cells treated with the negative control mimic and wildtype sequence vector. P-values are indicated as follows: * $p < 0.05$, ** $p < 0.01$, *** $p < 0.001$, **** $p < 0.0001$. ns- not significant

Supplemental Figure S6.related to Fig 4

AGO2 western blot analysis. Western blot images corresponding to Figure 4g, showing all replicates and respective loading control. (n=4)

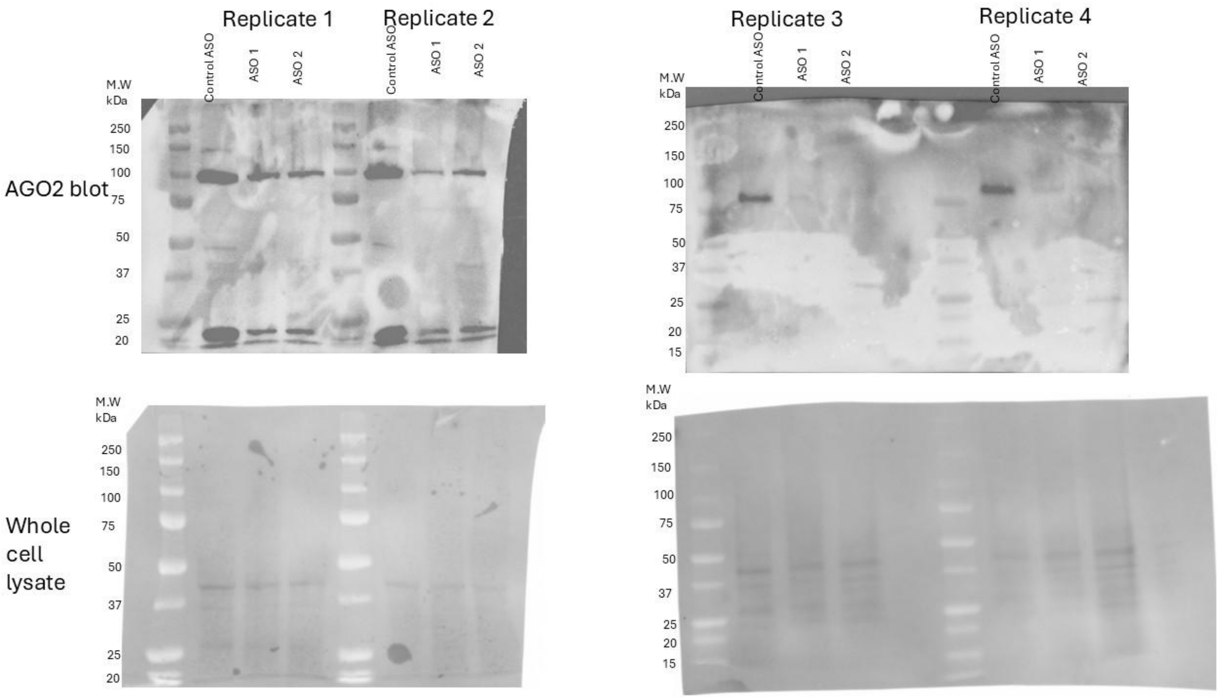

Supplemental Figure S7.related to Fig 4

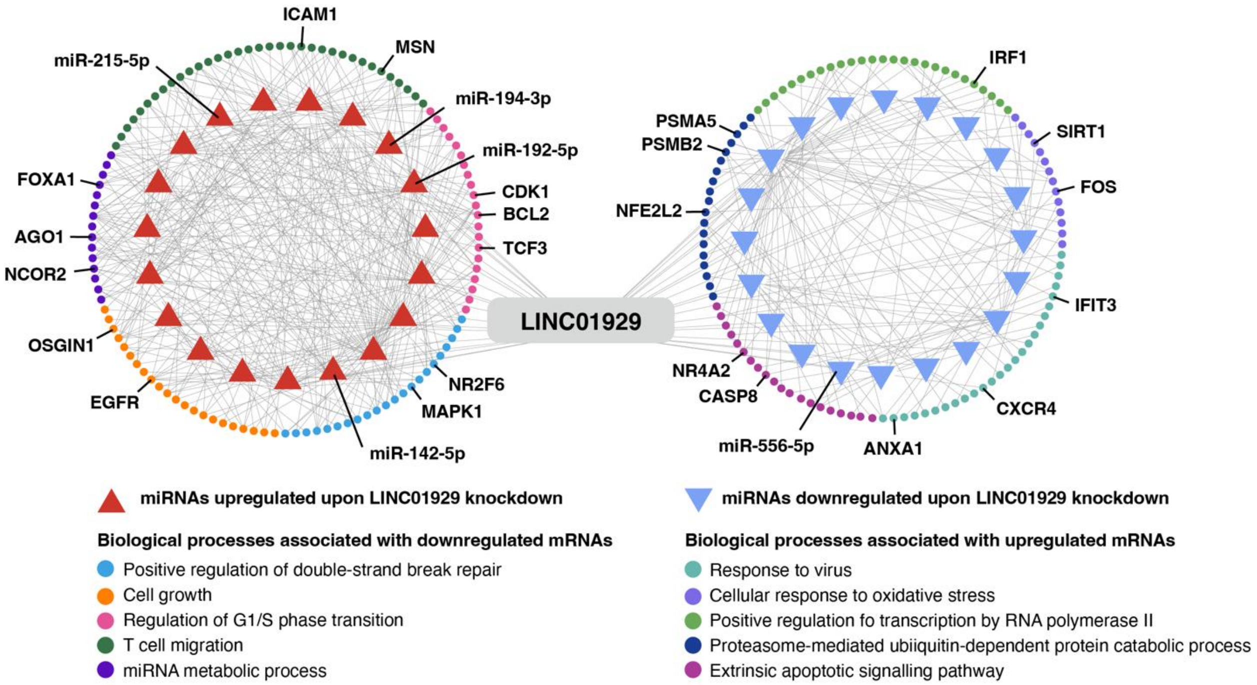

Predicted LINC01929-miRNA-mRNA interaction network in MDA-MB-231 cells. The network was generated using Cytoscape to visualize interactions between LINC01929, miRNAs, and mRNAs. mRNAs are color-coded based on their association with relevant biological processes.

Supplemental Figure S8.related to Fig 4

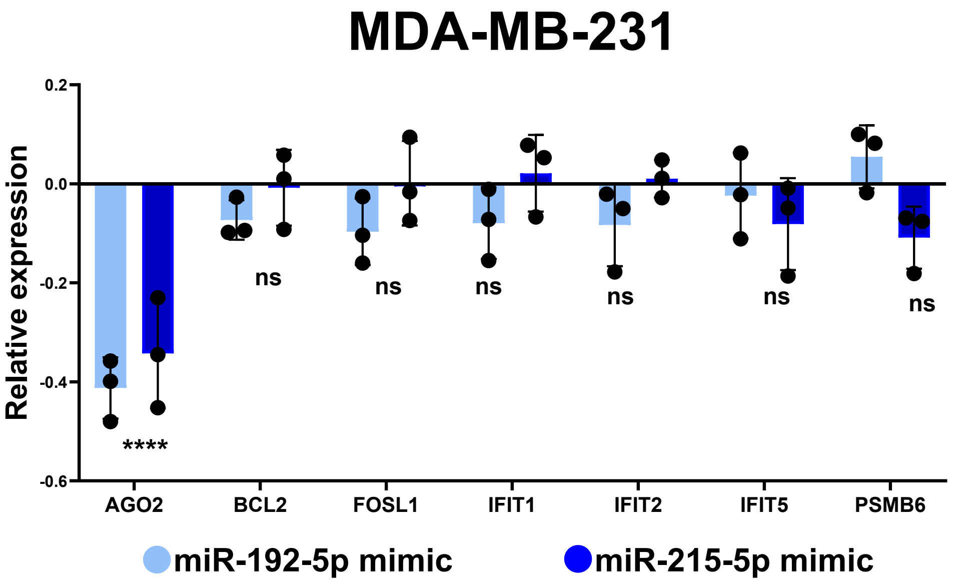

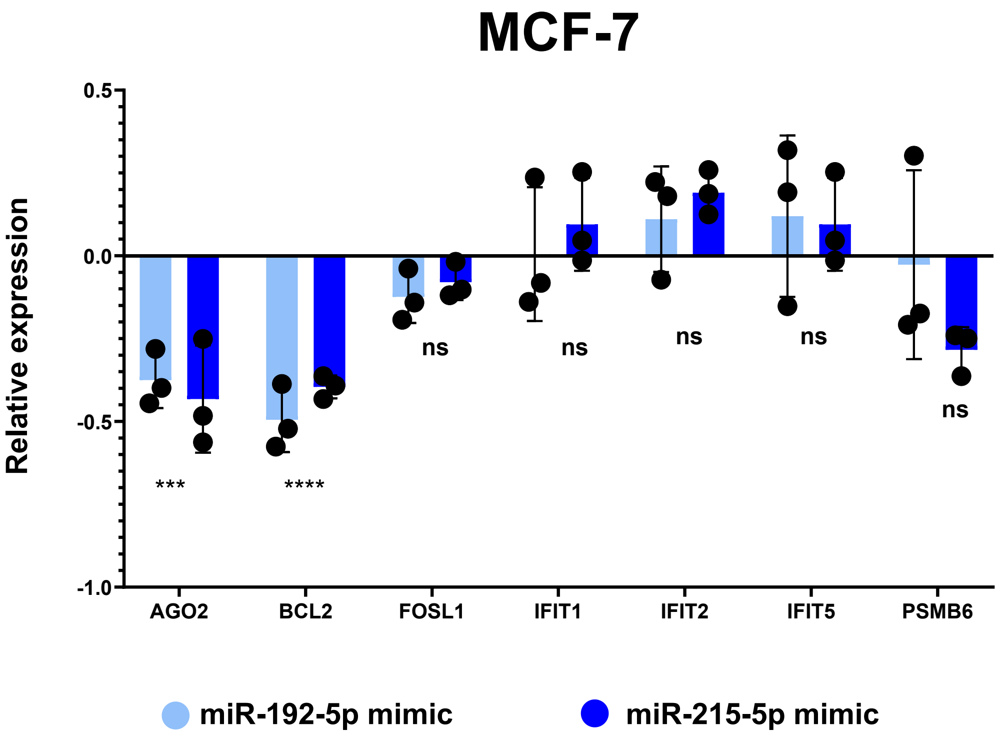

Transcriptional effect of miRNA mimics on LINC01929 regulated genes. qPCR of LINC01929 regulated genes treated with miR-192-5p (light blue) and miR-215-5p (dark blue) in MDA-MB-231 (top) and MCF-7 (bottom) breast cancer cells. Data represents relative expression in mimic treated cells compared to negative control microRNA mimic (N=3). Statistical significance was determined using a one-way ANOVA followed by Dunnett's multiple comparisons test. Error bars represent the standard deviation. P-values are indicated as follows: * $p < 0.05$, ** $p < 0.01$, *** $p < 0.001$, **** $p < 0.0001$. ns- not significant

Supplemental Figure S9. related to Fig 5

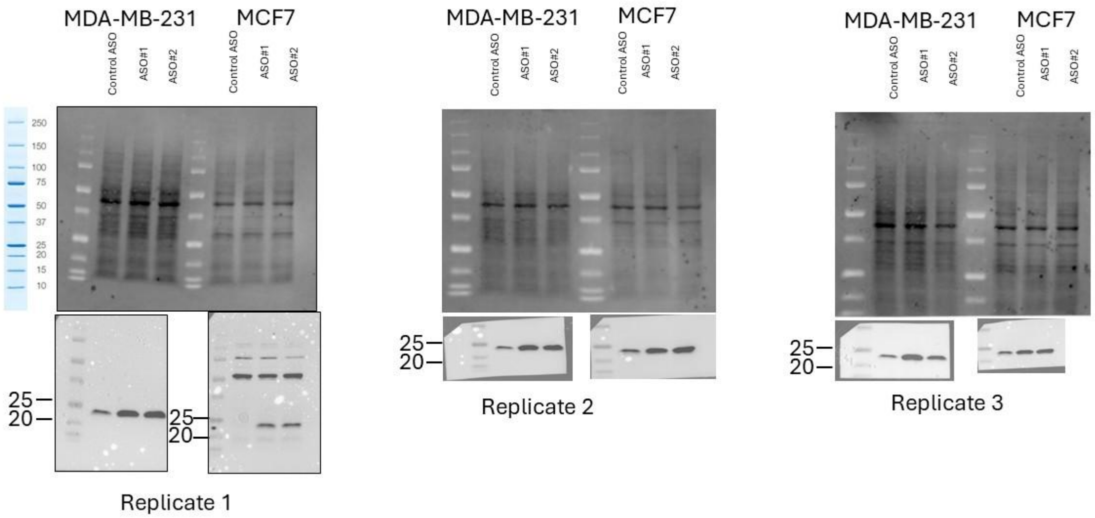

PSMB8 western blot analysis. Full western blot images corresponding to Figure 5c, showing total protein (top) and PSMB8 protein levels (bottom) for all replicates.

Supplemental Figure S10.related to Fig 6

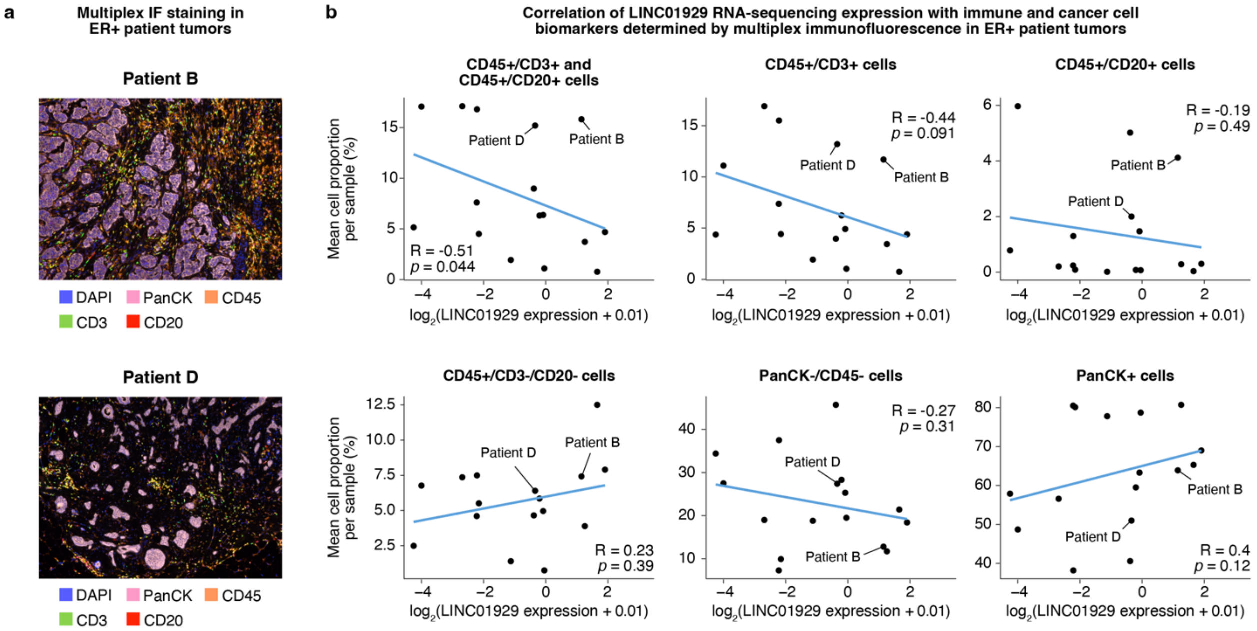

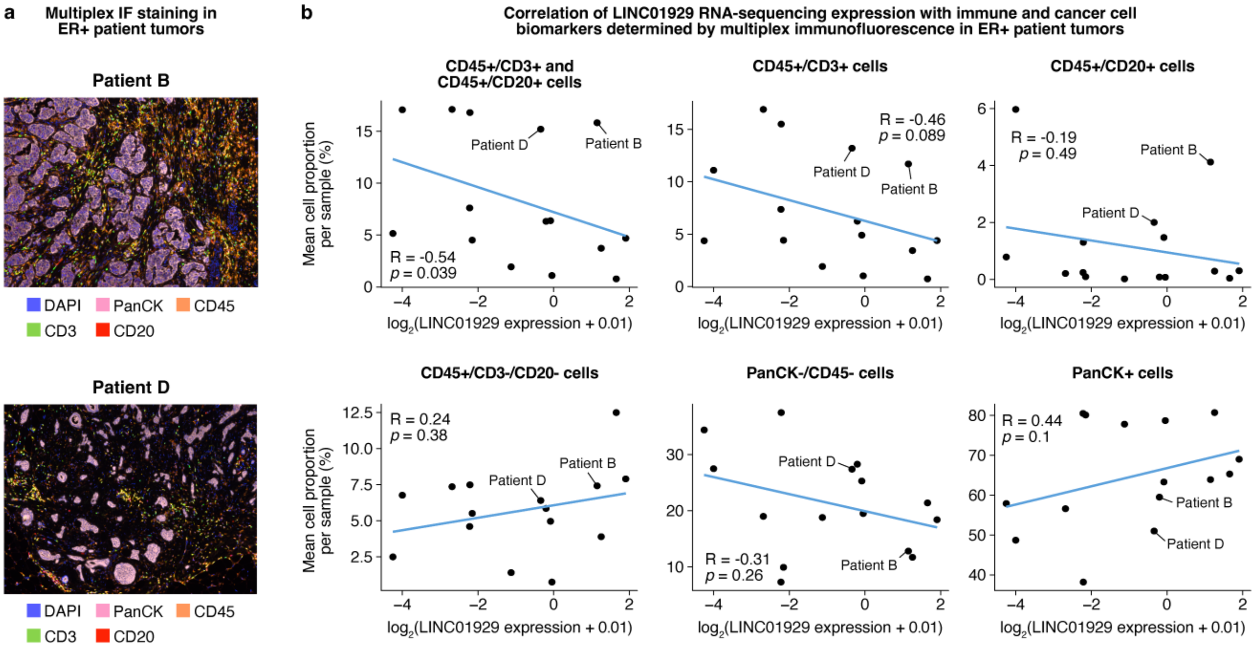

Correlation of LINC01929 expression with immune and cancer cell biomarkers in ER+ breast cancer patients. (a) Representative images of ER+ tumors with high (Patient B) and low (Patient D) immune cell infiltration. (b) RNA-sequencing data from the same ER+ patient samples were used to correlate LINC01929 expression with immune and cancer cell proportions in tumors. The Spearman correlation coefficient (R) and corresponding p-value are reported.

Supplemental Figure S11.related to Fig 6

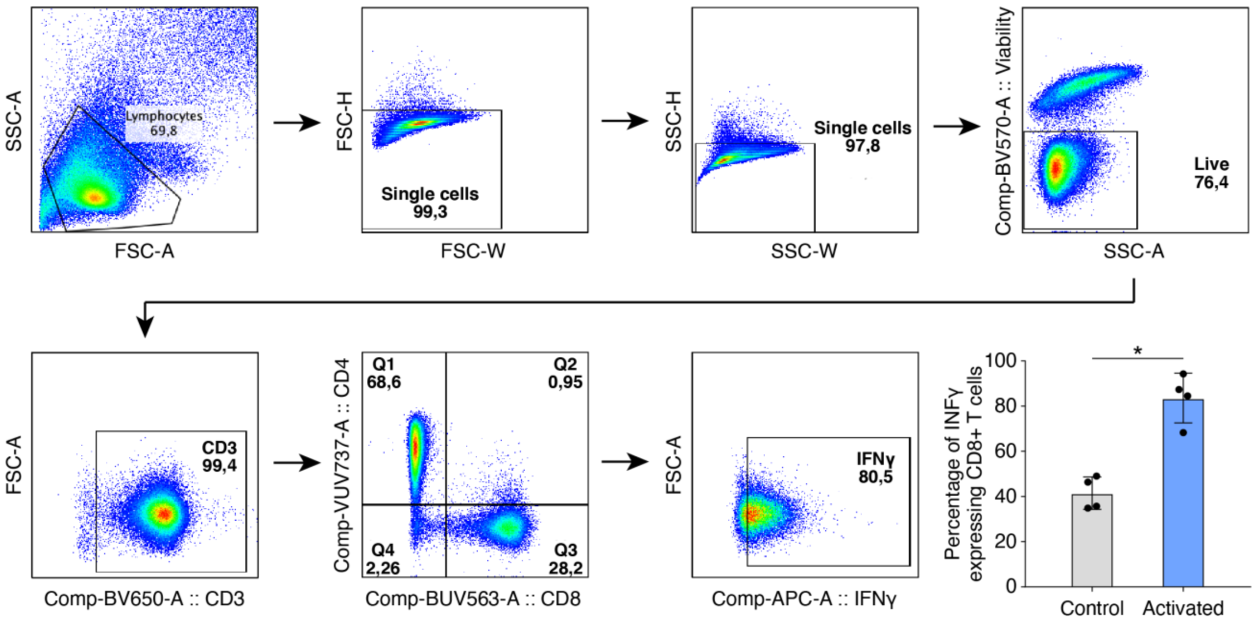

Validation of T cell activation in PBMCS cultured with CD3/CD28 beads. FACS gating scheme for measuring IFNγ in PBMCs activated with CD3/CD28 coated beads. Viable single cells are gated for CD3 and further gated for CD4 and CD8. IFNγ levels are measured in CD8+ T cells in the population and compared between control and CD3/CD28 activated cells. Experiment performed in N=4 donor PBMCs. Statistical analysis performed using paired t-test. p-values are indicated as: * = $p < 0.05$.

Supplemental Table S1. Related to Methods section

Target	ASO#	Catalogue #	Gene Globe ID	Sequence (5'-3')
Negative Control	N/A	339515	LG00000002	AACACGTCTATACGC
LINC01929	ASO1	339511	LG00788695	GTCGTGTCAAGTCATC
	ASO2	339511	LG00788862	CCATAGAAGAATTACT

Antisense oligonucleotide sequences

Supplemental Table S2. Related to Methods section

TCGA cohort	Disease name	Number of samples	
		Tumor	Normal-adjacent
**BLCA**	Bladder urothelial carcinoma	252	19
**BRCA**	Breast adenocarcinoma	837	105
**CESC**	Cervical squamous cell carcinoma and endocervical adenocarcinoma	196	3
**GBM**	Glioblastoma multiform	154	0
**HNSC**	Head and neck squamous cell carcinoma	426	42
**KICH**	Kidney chromophobe	66	25
**KIRC**	Kidney renal clear cell carcinoma	448	67
**KIRP**	Kidney renal papillary cell carcinoma	198	30
**LGG**	Brain lower grade glioma	486	0
**LIHC**	Liver hepatocellular carcinoma	200	50
**LUAD**	Lung adenocarcinoma	488	58
**LUSC**	Lung squamous cell carcinoma	220	17
**OV**	Ovarian squamous cystadenocarcinoma	412	0
**PRAD**	Prostate adenocarcinoma	374	52
**SKCM**	Skin cutaneous melanoma	226	0
**STAD**	Stomach adenocarcinoma	285	33
**THCA**	Thyroid carcinoma	497	59

TCGA cohorts accessed

Supplemental Table S3. Related to methods section

Cell line	Source	Media	FBS	Antibiotic/ Antimycotic	Human Insulin	Conditions
MCF7	ATCC	DMEM	10%	1%	-	5% CO2, 37^{0}C
T-47D	ATCC	DMEM	10%	1%	-	5% CO2, 37^{0}C
MDA-MB-468	ATCC	DMEM	10%	1%	-	5% CO2, 37^{0}C
MDA-MB-231	ATCC	DMEM	10%	1%	-	5% CO2, 37^{0}C
SKBR3	ATCC	DMEM	10%	1%	-	5% CO2, 37^{0}C
MCF-10A	ATCC	DMEM	20%	1%	-	5% CO2, 37^{0}C
Hs578Bst	ATCC	DMEM	10%	1%	10 µg/mL	5% CO2, 37^{0}C

Cell line culture conditions

Supplemental Table S4. Related to methods section

Gene	Forward Primer (5'-3')	Reverse primer (5'-3')
AGO2	CCATCCGTGAGGCCTGTATC	CCTCTTCTGCACCACGATGA
ARF1	GTGTTCGCCAACAAGCAGG	CAGTTCCTGTGGCGTAGTGA
B2M	AGGCTATCCAGCGTACTCCA	CGGATGGATGAAACCCAGACA
BCL2	GGGGTCATGTGTGTGGAGAG	GCCGGTTCAGGTACTCAGTC
CASP8	GCTGACTTTCTGCTGGGGAT	GACATCGCTCTCTCAGGCTC
CDK1	GTGCTTATGCAGGATTCCAGGT	ATCCATGTACTGACCAGGAGGG
CUL5	TGGACGTTTGCAGCTCACTA	TGTATGATAGCTTCCTGGGTTCTT
DANCR	AGGAGTTCGTCTCTTACGTCT	TGAAATACCAGCAACAGGACA
FOSL1	CTGGTGCCAAGCATCAACAC	TGCAGGAAGTCGGTCAGTTC
GAPDH-Human	GGAGTCAACGGATTTGGTCGTA	TTCTCCATGGTGGTGAAGAC
GAPDH-Mouse	CGCCCCACTTGATTTTGGAG	GGCGGAGATGATGACCCTTT
HPRT1	GACCAGTCAACAGGGGACAT	CCTGACCAAGGAAAGCAAAG
HSPA4	AGATTCCATGGCCGAGCATT	TCCTGTAGGCAACTGCACAAT
IFIT1	GATCTCTGCCTATCGCCTGG	TGCCTTAGGGGAAGCAAAGAA
IFIT2	CCTCTGGACTGGCAATAGCA	AGAGGGTCAATGGCGTTCTG
IFIT5	TACGCTGAAGGAGGCCAGTA	CTGAAAGCGGCCATAGTGGT
JADE1	TCCGAAGAAGGGTGGAGCTA	CAGAGCACAGCTAACGTGGA
LINC01929	TATTCTGGCCCATCGTGGC	CTGAAGCAGAGCTCGACCAG
LINC01929 -2nd primer	TCCTCTCATACCACTAACATC	GCACCAACTTCAAGACAAT

MCM8	GGCTATGCTCGGCAGTATGT	AACCTCTGGCTCTGTTTCCG
MLTT11	CCCAGAACTGGATCTGTCGG	TGCTTGCCCGATCATTTTGC
NEAT1	CCTCCCTTTAACTTATCCATTCAC	TCTCTTCCTCCACCATTACCA
PSMA1	ATGCGTCAGGAGTGTTTGGA	CCGGCCATATCGTTGTGTTG
PSMA5	TGCCATGTCTCGTCCCTTTG	ATGAAACAGCTGGGGTCCTTT
PSMB4	TTACCGCATTCCGTCCACTC	CGCGTGATTGGACCTCTGTA
PSMB6	ACTGCCAATGCTCTCGCTT	TACTTGCCGCTCTACCCCT
PSMC1	GGACCCCGATGTCAGTAGGA	GCCCACAGATGTAGACACGA
PUM1	GGCGTTAGCATGGTGGAGTA	CATCCCTTGGGCCAAATCCT
TBP	GGCACCACTCCACTGTATCC	GCTGCGGTACAATCCCAGAA

Primer sequences

Supplemental Table S5. Related to methods section

Target	Catalogue #	5’ Sequence	3’ Sequence
Negative Control	TLNSU4433	5’ Sequence	AAGGCAGAAGTATGCAAAGCAT
		3’ Sequence	ATGCTTTGCATACTTCTGCCTG
		Loop Sequence	TAGTGAAGCCACAGATGTA
LINC01929	ULTRA-37463979	5’ Sequence	AGAGGTGGACAGAAGTGATATA
		3’ Sequence	ATATATCACTTCTGTCCACCT
		Loop Sequence	TAGTGAAGCCACAGATGTA

LINC01929 shRNA sequences

Supplemental Table S6. Related to methods section

Oligo name	Sequence (5’-3’)
LINC01929 WT F	GCTGAGATATCTAGGTCA
LINC01929 WT R	TCGATGACCTAGATATCTCAGC
MUT F	CTTAGCATATGCACGACG
MUT R	TCGACGTCGTGCATATGCTAAG

LINC01929-miR-192-5p and miR-215-5p binding sequences and their mutated oligos
